# Reassessment of the *Listeria monocytogenes* pan-genome reveals dynamic integration hotspots and mobile genetic elements as major components of the accessory genome

**DOI:** 10.1186/1471-2164-14-47

**Published:** 2013-01-22

**Authors:** Carsten Kuenne, André Billion, Mobarak Abu Mraheil, Axel Strittmatter, Rolf Daniel, Alexander Goesmann, Sukhadeo Barbuddhe, Torsten Hain, Trinad Chakraborty

**Affiliations:** 1 Institute of Medical Microbiology, German Centre for Infection Research, Justus-Liebig-University, D-35392, Giessen, Germany; 2 Department of Genomic and Applied Microbiology and Goettingen Genomics Laboratory, Institute of Microbiology and Genetics, Georg-August University Goettingen, Grisebachstrasse 8, D-37077, Goettingen, Germany; 3 Bioinformatics Resource Facility, Center for Biotechnology, Bielefeld University, D-33549, Bielefeld, Germany; 4 ICAR Research Complex for Goa, Ela, Old Goa, 403402, India

## Abstract

**Background:**

*Listeria monocytogenes* is an important food-borne pathogen and model organism for host-pathogen interaction, thus representing an invaluable target considering research on the forces governing the evolution of such microbes. The diversity of this species has not been exhaustively explored yet, as previous efforts have focused on analyses of serotypes primarily implicated in human listeriosis. We conducted complete genome sequencing of 11 strains employing 454 GS FLX technology, thereby achieving full coverage of all serotypes including the first complete strains of serotypes 1/2b, 3c, 3b, 4c, 4d, and 4e. These were comparatively analyzed in conjunction with publicly available data and assessed for pathogenicity in the *Galleria mellonella* insect model.

**Results:**

The species pan-genome of *L. monocytogenes* is highly stable but open, suggesting an ability to adapt to new niches by generating or including new genetic information. The majority of gene-scale differences represented by the accessory genome resulted from nine hyper variable hotspots, a similar number of different prophages, three transposons (Tn916, Tn554, IS3-like), and two mobilizable islands. Only a subset of strains showed CRISPR/Cas bacteriophage resistance systems of different subtypes, suggesting a supplementary function in maintenance of chromosomal stability. Multiple phylogenetic branches of the genus *Listeria* imply long common histories of strains of each lineage as revealed by a SNP-based core genome tree highlighting the impact of small mutations for the evolution of species *L. monocytogenes*. Frequent loss or truncation of genes described to be vital for virulence or pathogenicity was confirmed as a recurring pattern, especially for strains belonging to lineages III and II. New candidate genes implicated in virulence function were predicted based on functional domains and phylogenetic distribution. A comparative analysis of small regulatory RNA candidates supports observations of a differential distribution of *trans*-encoded RNA, hinting at a diverse range of adaptations and regulatory impact.

**Conclusions:**

This study determined commonly occurring hyper variable hotspots and mobile elements as primary effectors of quantitative gene-scale evolution of species *L. monocytogenes*, while gene decay and SNPs seem to represent major factors influencing long-term evolution. The discovery of common and disparately distributed genes considering lineages, serogroups, serotypes and strains of species *L. monocytogenes* will assist in diagnostic, phylogenetic and functional research, supported by the comparative genomic GECO-LisDB analysis server (http://bioinfo.mikrobio.med.uni-giessen.de/geco2lisdb).

## Background

The genus *Listeria* consists of eight species being *L. monocytogenes*, *L. innocua*, *L. welshimeri*, *L. seeligeri*, *L. ivanovii*, *L. grayi*, *L. marthii* and *L. rocourtiae*[1-3]. *Listeria* are saprotrophic with *L. monocytogenes* and *L. ivanovii* considered facultative pathogens, the latter predominantly causing infections in ruminants [4]. *L. monocytogenes* represents the species most commonly associated with listeriosis in humans which primarily affects immunocompromised individuals [5]. The majority of infections are thought to be foodborne and results in high mortality rates [6].

Strains of *L. monocytogenes* can be grouped into four evolutionary lineages and 12 serotypes representing distinct phylogenetic, ecologic and phenotypic characteristics [7-9]. Lineage I was found to be overrepresented among human clinical isolates and epidemic outbreaks in most studies while lineage II is typically sporadically isolated from both humans and animals. Lineage III and IV are rare and predominantly identified in animals. These associations show frequent regional differences, thus rendering the definition of a natural environment difficult. Lineages II, III and IV show higher recombination rates and a lower degree of sequence similarity than lineage I. This observation was proposed to result from less diverse lifestyles for the latter and may denote strains of lineage I as descendants of a recently emerged highly virulent clone [10,11]. Plasmids are more prevalent in lineage II and include a multitude of resistance genes dealing with toxic metals, horizontal gene transfer, oxidative stress and small toxic peptides [12]. Furthermore, strains of this lineage often show virulence attenuated phenotypes due to deletions inside important virulence genes [13]. About 98% of human cases of listeriosis are caused by strains of serotypes 4b, 1/2a, 1/2b and 1/2c [14].

Virulence of the bacterium is heavily dependent on the virulence gene cluster (VGC, LIPI-1) which promotes cytosolic replication as well as intra- and intercellular movement [15]. A second cluster required for virulence contains an operon of two genes (*inlA/B*) that encode internalins necessary for the attachment to and invasion of non-phagocytic host cells [16]. The species *L. ivanovii* displays a specific island with virulence factors called LIPI-2, comprising of multiple internalins and *smcL* sphingomyelinase hemolysis gene [17]. A subset of strains of lineage I carry an additional hemolysin called listeriolysin S (LIPI-3) which contributes to virulence *in vitro*[18]. Other genes involved in the infectious process modulate the bacterial metabolism and stress response [19,20]. Interestingly, prophage genes may also have a function in virulence as identified by transcriptomic analyses of intracellular regulation of genes of three major lineages [21].

A variety of cell wall components are important for the survival of strains of species *L. monocytogenes* in the environment and the infected host, which are frequently encoded by genes harboring domains involved in cell-wall anchoring or protein-protein interactions (e.g. LPXTG, GW, P60, LysM, lipo-box, LRR) [9,22-26].

To protect from bacteriophage activity, some Archaea and bacteria have developed an adaptive immune system (CRISPR: clustered regularly interspaced short palindromic repeats) based on a variable module of repeats, spacers and protein coding genes (Cas: CRISPR associated) [27]. Recently it was shown that CRISPR spacers can bear sequences homologous to chromosomal genes which may represent a form of autoimmunity or regulatory mechanism [28,29]. Some CRISPR/Cas subtypes lacking endoribonucleases necessary for the maturation of crRNAs were shown to appropriate a *trans*-encoded small RNA (*tracrRNA*) in combination with a host factor (RNase III) in order to facilitate the silencing of foreign nucleic acids [30]. CRISPR/Cas systems were previously identified inside a number of strains of genus *Listeria* but never discussed in detail [21,30-32].

Small non-coding regulatory RNAs have emerged as a further layer of gene expression regulation in prokaryotes [33]. They regulate transcription by pairing with other RNAs, forming parts of RNA-protein complexes, or adopting regulatory secondary structures [34]. Small non-coding RNAs were previously identified in species *L. monocytogenes* based on microarrays or deep sequencing approaches and have been implicated in responses to iron limitation, oxidative stress, low temperature and intracellular growth [35-41].

The pan-genome concept has recently been introduced to explore the diversity of a number of bacterial species and found varying degrees of conservation reflecting differences in habitat, evolutionary pressure and gene pool [42-46]. Analyses of the pan-genome of genus *Listeria* showed that gene loss played an important role in the development of modern *Listeria* species from a putatively pathogenic ancestor [31]. Previous attempts to study the pan-genome of *L. monocytogenes* were focused on the identification of genes present in lineage I/II while being absent in lineage III and based on microarrays containing mostly draft quality genomes missing several serotypes, thus limiting the possible resolution [9,47].

This study is the first one to base its evolutionary analyses on a set of 16 completely sequenced genomes of species *L. monocytogenes* including strains of all serotypes, arguably bearing the most diverse pan-genome to be assessed for this species. These include five previously sequenced and extensively studied strains of three major lineages (I-III) being 4a L99, 4b F2365, 1/2a EGD-e, 1/2a 08–5578 and 1/2a 08–5923 as well as the eleven newly sequenced genomes [21,32,48,49]. Efficient invasion into epithelial cells was described for strains 1/2a EGD-e, 1/2b SLCC2755, and 4b L312 while strains 4c SLCC2376, 3a SLCC7179, 3c SLCC2479, 1/2c SLCC2372, 7 SLCC2482, 3b SLCC2540, 4e SLCC2378, and 4d ATCC19117 displayed attenuation or absence of this ability [50]. An association with human illness was previously established for strains 1/2c SLCC2372, 1/2a 08-5923/08-5578, 3b SLCC2540, and 4b F2365 [32,49,51,52]. We determined common and distinct genetic elements to understand the diversity of forces shaping the species down to the level of strains. Most of the analyses were conducted using the GECO comparative genomics software, which was heavily extended in relation to the previously published version in order to satisfy the needs of this study [53]. This work focuses on major molecular aspects relating to evolutionary adaptation of species *L. monocytogenes*, and is intended to serve as a framework to support future analyses for the *Listeria* research community.

## Results and discussion

### Basic features of strains selected among known serotypes of *L. monocytogenes*

In order to analyze the evolution and pan-genomic potential of the species, strains of *L. monocytogenes* spanning all known serotypes originating from various sources were selected for comparison (Table 1). The chromosome of *L. monocytogenes* 7 SLCC2482 contains one gap located at 2125011 bp and estimated to have a size of approximately 10000 bp. Four strains harbored plasmids which were described previously [12]. All strains were classified according to known sequence types and chromosomal complexes using the BIGSdb software [7,54].

**Table 1:** **Origin of compared strains of species *L. monocytogenes***

**Serotype**	**Strain**	**Lineage**	**Chromosome accession**	**Plasmid accession**	ST*	CC*	**Source of isolate**	**Year of isolation**	**Country of isolation**	**Reference**
4c	SLCC 2376	III	FR733651		71		poultry			SLCC: Haase et al. (2011)
4a	L99	III	FM211688		201		cheese	1950	Netherlands	Hain et al. (2012)
3a	SLCC 7179	II	FR733650		91		cheese	1986	Austria	SLCC: Haase et al. (2011)
3c	SLCC 2479	II	FR733649		9	9		1966		SLCC: Haase et al. (2011)
1/2c	SLCC 2372	II	FR733648	FR667691	122	9	human	1935	UK	SLCC: Haase et al. (2011)
1/2a	08-5923	II	NC_013768		120		human	2008	Canada	Gilmour et al. (2010)
1/2a	08-5578	II	NC_013766	CP001603			human	2008	Canada	Gilmour et al. (2010)
1/2a	SLCC 5850	II	FR733647		12	7	rabbit	1924	UK	SLCC: Haase et al. (2011)
1/2a	EGD-e	II	NC_003210		35	9	rabbit	1926	UK	Glaser et al. (2001)
7	SLCC 2482	I	FR720325	FR667690	3	3	human	1966		SLCC: Haase et al. (2011)
1/2b	SLCC 2755	I	FR733646	FR667692	66	3	chinchilla	1967		SLCC: Haase et al. (2011)
3b	SLCC 2540	I	FR733645				human	1956	USA	SLCC: Haase et al. (2011)
4e	SLCC 2378	I	FR733644		73	1	poultry			SLCC: Haase et al. (2011)
4d	ATCC 19117	I	FR733643		2	2	sheep			SLCC: Haase et al. (2011)
4b	L312	I	FR733642		4	4	cheese			Chatterjee et al. (2006)
4b	F2365	I	NC_002973		1	1	cheese	1985	USA	Nelson et al. (2004)

*Sequence Type.

**Clonal Complex.

The chromosomes compared show a similar size, G+C content, average length of protein coding genes and percentage of protein coding DNA (Table 2). The number of coding sequences ranged from 2755 (SLCC2376) to 3010 (08–5578). We identified six 16S-23S-5S-rRNA operons in most strains with the exception of 1/2a 08–5578 and 1/2a 08–5923 which lack one rRNA module and several tRNAs.

**Table 2:** General features of the chromosomes of compared strains

**Strain**	**Gaps**	**Length of chromosome[bp]**	**G+C content[%]**	**Number of CDS**	**Protein coding DNA[%]**	**Number of rRNA genes**	**Number of tRNA genes**
SLCC 2376	closed	2840185	38.3	2755	89.3	18	67
L99	closed	2979198	38.2	2925	88.9	18	67
SLCC 7179	closed	2882234	38.0	2826	89.3	18	67
SLCC 2479	closed	2972172	38.0	2935	89.3	18	65
SLCC 2372	closed	2972810	38.0	2936	89.3	18	67
08-5923	closed	2999054	38.0	2966	89.3	15	58
08-5578	closed	3032288	38.0	3010	89.3	15	58
SLCC 5850	closed	2907142	38.0	2866	89.2	18	67
EGD-e	closed	2944528	38.0	2855	89.2	18	67
SLCC 2482	1	2936689*	38.0	2874	89.1	18	67
SLCC 2755	closed	2966146	38.1	2877	89.3	18	67
SLCC 2540	closed	2976958	37.9	2907	89.4	18	67
SLCC 2378	closed	2941360	38.0	2874	89.1	18	66
ATCC 19117	closed	2951805	38.0	2868	89.3	18	67
L312	closed	2912346	38.1	2821	89.3	18	67
F2365	closed	2905187	38.0	2847	88.4	18	67

*including 100 N gap spacer.

### Pan-genome model predicts a highly conserved species

**Figure 1:**
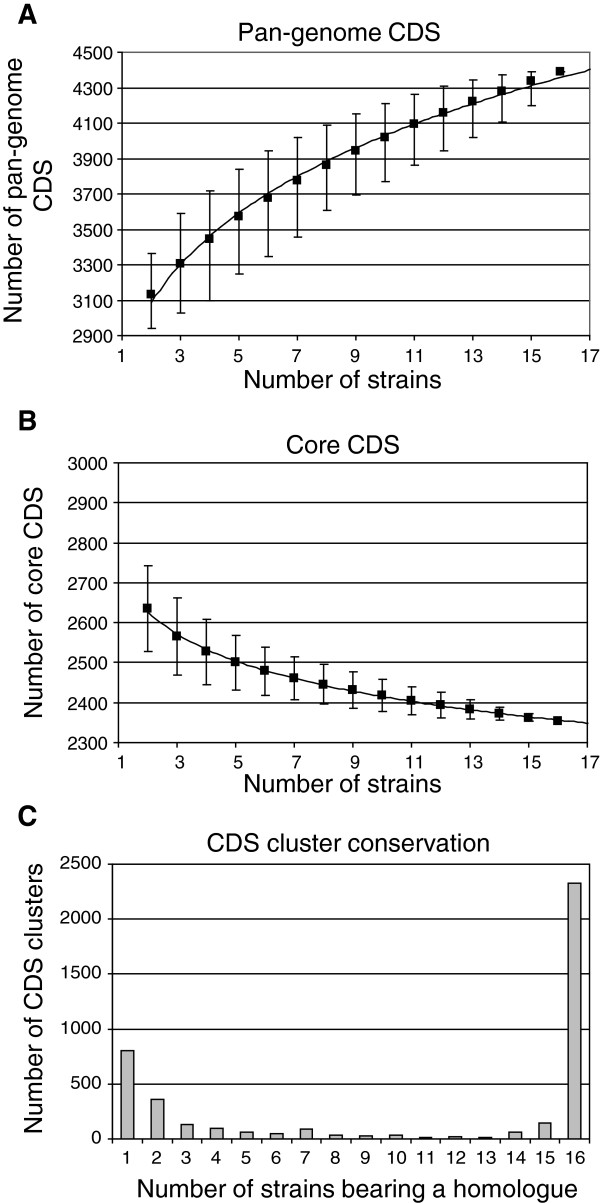
**Pan-genomic distribution.** Distribution of CDS based on a homology measure of 60% amino acid identity and 80% coverage. Chromosomes were added 10000 times without replacement in a randomized order and the number of core (mutually conserved) and accessory (found in at least one but not all strains) genes was noted. Since mean and median values for each step showed only little variation the mean numbers of gene classes were plotted. In order to predict a possible future pan-genomic distribution for this species we performed a power law fitting. **A**) Pan-genomic CDS after each consecutive addition of a strain, **B**) mutually conserved CDS, **C**) conservation of CDS and homology clusters.

The pan-genome of 16 chromosomes of *L. monocytogenes* was found to contain 4387 genes including 114 paralogues based on a similarity cutoff of 60% amino acid identity and 80% coverage of protein alignments (Figure 1). Approximately 78% of coding sequences per strain consist of mutually conserved core genes (2354 / species) indicating a highly stable species backbone with relatively few accessory genes (2033 / species) (Additional file 1). More than half of the species accessory genes (1161) furthermore displayed homologues in only one or two strains implying relatively recent insertions that are rarely fixed in the population. A power law regression analysis predicting a future pan-genomic distribution after further sequencing resulted in a mean power law fitting for new genes of n=397.4N^-0.7279^ (α=0.7279). This indicates a conserved but open pan-genome that permits limited integration of foreign DNA or generation of genetic diversity by other evolutionary forces such as mutation, duplication and recombination as previously described [55]. Regression curves predict the presence of ca. 6000 different genes in the pan-genome of *L. monocytogenes* after 100 strains have been completely sequenced.

Other studies relying on the hybridization of eight lineage III strains on a microarray based on 20 strains (two complete, 18 draft chromosomes) found a closed species pan-genome [47,56]. These likely represent an underestimation of true sequence diversity of the species because they lack multiple serotypes (e.g. 3a, 3b, 3c, 4e, 7), less stringent similarity cutoffs and a lower number of fully sequenced strains. The pan-genome of genus *Listeria* based on chromosomes of 13 strains (six complete, seven draft) was determined to be open [31].

Summarily, our research shows a conserved species, which tolerates low levels of horizontal gene transfer.

### Hyper variable hotspots contain one fourth of the accessory genes and permitted the insertion of major pathogenicity determinants

The accessory gene content of compared strains is not scattered evenly across the chromosomes, but accumulates in nine defined chromosomal regions supporting previous observations considering the clustered distribution of strain-specific genes [57] (Additional file 2, Additional file 3). These hotspots were defined by the localization of at least three non-homologous insertions between mutually conserved core genes. The latter showed no over representation among any particular functional or genetic category. Nearly every fourth of the accessory genes (454 = 22%) was found to be located in such a highly variable region. Interestingly, strains of lineage III displayed an average of 56 genes inside these loci, while strains of lineage I and II contained nearly twice as many (80–90), indicating either stronger deleterious forces in the former or an increased number of insertions in the latter. One third of these genes were accounted for by strain-specific insertions leading to a low average conservation of hotspot genes in only three strains. The majority of these genes have no known function (298), 35 are part of restriction modification systems, and 13 are involved in genetic mobilization.

Only a small number of genes could be identified inside hotspot loci which exhibit an obvious adaptive value for the host genome, including the previously described pathogenicity determinants *inlA/B* and LIPI-3 [16,18]. Transposon Tn916 introduced additional cadmium resistance genes into its host strain 1/2a EGD-e [19]. Two variants of an IS3-like transposon were inserted in different hotspot integration sites of the epidemic lineage I and found to bear multiple surface-associated proteins. The latter are implied in attachment, invasion, and other interactions with the environment and were identified in most hotspots resulting in the presence of a total of 40 genes of this category.

These hyper variable hotspots have previously been suggested to be the result of a founder effect resulting from a primary insertion that did not reduce the fitness of the respective strain, which now offers a larger target for neutral insertions, thus increasing their likelihood [44]. It is tempting to speculate, that these regions represent evolutionary test areas attracting new genetic information by frequent insertions, deletions and other differentiating forces, rarely leading to fixation of genes in the population. Interestingly, all but one of these hotspots are located on the right replichore, which thus represents an area of increased genomic plasticity. Only half of the variable regions displayed identifiable mobilization genes indicating either unidentified mobilization genes, decay or other means to facilitate insertions putatively including also mechanisms for homologous recombination.

### Chromosomal mobile genetic elements are major sources of diversity – prophages, transposons and genetic islands

**Figure 2:**
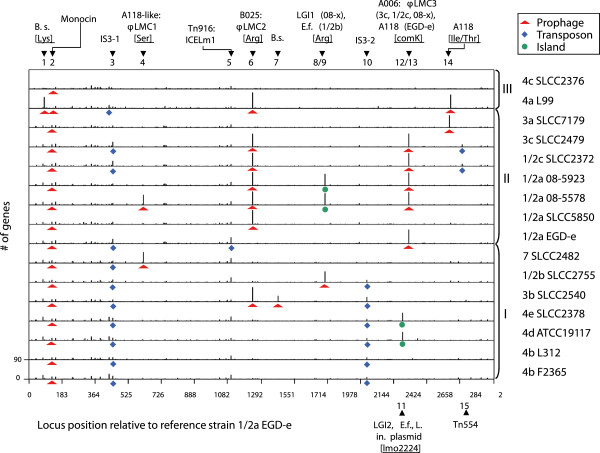
**Insertions between syntenic core genes.** Bar chart of CDS inserted between syntenic core-CDS existing in all strains depicted relative to reference strain 1/2a EGD-e. The oriC inversion of strains 08–5923 and 08–5578 was removed for this analysis. Mobile genetic elements (MGE) are classified as prophage (red triangle), transposon/IS element (blue square), genetic island (green circle). The MGEs were numbered according to their relative position in strain 1/2a EGD-e. Putative anchor genes in the chromosome (ex.: tRNA, comK) are included in square brackets. If different elements inserted at the same chromosomal locus, the strains involved are denoted in round brackets. Multiple designations per element are delimited by a colon. If an element was not described yet, the genus bearing the highest overall nucleotide similarity to the respective region was included instead (e.g. *Bacillus subtilis, Enterococcus faecalis*). Lineages are denoted with roman numbers.

In order to find large insertions in the chromosomes of the respective strains we plotted all coding sequences, which were not conserved in all strains, resulting in the identification of between one and five mobile genetic elements (MGE) such as prophages, transposons, insertion sequences and genomic islands per chromosome (Figure 2). These introduced 6 to 235 protein coding genes per strain included in 15 different MGE insertions into 13 distinct chromosomal loci (Additional file 4). This translates into 703 genes of the pan-genome (15%) or one third of the accessory genes.

Among these are 8 different prophages which are typically inserted by site-specific recombination into chromosomal loci adjacent to tRNA genes as previously observed [58]. We also found two different bacteriophages (A006 and A118) which targeted the *comK* gene [59]. Most prophages belong to the class of listeriaphages (B025, A118, and A006) or show a high similarity to unnamed prophages also found in the genera *Bacillus, Enterococcus, Clostridium* and *Staphylococcus*. It should be noted that the only strains without apparently complete prophages are both strains of serotype 4b as well as 4c SLCC2376. Rarity of prophages in serogroup 4 was previously proposed to result from differences in teichoic acid composition, which is supported by strains of this study due to the absence of 12 out of 16 genes of an operon encoding a rhamnose pathway for teichoic acid biosynthesis conserved in all other compared strains (*lmo1076-lmo1091*) as well as several missing glycosyl transferases (*lmo0497, lmo0933, lmo2550*) [60].

Three putative transposons were identified in the strains studied. Two of them are located between homologues of genes *lmo1096-lmo1115* in strain EGD-e (ICELm1, TN916-like) and *lmo2676-lmo2677* in 3c SLCC2479 and 1/2c SLCC2372 (TN554-like), respectively (Additional file 5) [61]. ICELm1 contains two genes involved in cadmium resistance and a fibrinogen-binding protein with an LPXTG domain which is implied in host cell attachment in *Staphylococcus epidermidis*[62]. The Tn554-like transposon introduced an arsenate resistance operon (*arsCBADR*) also found in *Enterococcus faecalis* (ca. 70% amino acid identity) into its host chromosomes. The third putative transposon consists of 15 genes including two insertion elements bearing two IS3-type transposases as found in its complete form in strain 3b SLCC2540 (Additional file 6). It contains a module consisting of a transcriptional regulator and four homologues of a lipoprotein. The latter was predicted by previous studies to furthermore contain an RGD motif implied in integrin binding and a weak homology to leucine-rich-repeat domains, indicating a putative function in host-pathogen interaction [23,48]. Deletion versions of this transposon, which have lost one insertion element, can be found at the same relative position at approximately 2.1 Mb (e.g. *LMOf2365_2051-9*) in all strains of lineage I and another variant at ca. 0.5 Mb (e.g. *LMOf2365_0493-500)* in a subset of strains of all lineages. Interestingly, indels of the complete transposon and the lipoprotein itself have led to a distribution of 4–7 instances of the lipoprotein in epidemic lineage I in comparison to 0–1 in lineages II and III, which further indicates these two modules as potential targets for research regarding virulence determinants. All but one transposon were found in a hyper variable hotspot suggesting either relaxed deleterious forces in these areas or an enrichment of repeats targeted by the respective mobilization genes.

Another type of MGE is designated genomic island and denotes a module of genes inserted by horizontal gene transfer which frequently encodes fitness conferring genes and typically contains at least one integrase gene employed for mobility. One of these was called Listeria genomic island 1 (LGI1) and putatively introduced by serine recombinases into 1/2a 08–5923 and 08–5578 [49]. It was described to include genes involved in secretion, protein-protein interaction, adhesion, multidrug efflux, signal transduction and restriction modification. We identified a second genomic island named LGI2, which has not yet been described in the literature. It spans approximately 35000 bp in strains 4e SLCC2378 and 4d ATCC19117 and integrated into genes orthologous to *lmo2224* (1/2a EGD-e). This mobile element consists of 36 genes and putatively inserted by means of a bacteriophage integrase (*LMOSLCC2378_2256*) distantly related to temperate *Lactococcus lactis* bacteriophage phiLC3 [63]. Additionally, a putative operon of eight genes coding for arsenate resistance proteins (*LMOSLCC2378_2263-70*) was found to be homologous to a region of *Listeria innocua* Clip11262 plasmid pLI100, indicating recombination between phages, plasmids and chromosomes which resulted in the formation of this mobile element. Other genes of this locus code for ATP transporters, a putative anti-restriction protein, a secreted and a cell wall surface anchor protein.

In summary, nearly one third of the accessory genes of the species have been introduced by identifiable MGEs, representing a large proportion of gene-scale diversity [64]. The distribution of most MGEs is heterogeneous indicating either recent insertions and/or frequent deletion of these sequences. Prophage-related genes of species *L. monocytogenes* represent major chromosomal disparities, have been described to assist intracellular survival, and were found to serve as genetic switches in order to modulate the virulence of its host [21,64-67]. The general rarity of mobile genetic elements in the compared strains nonetheless supposes mechanisms to limit inclusion of foreign DNA as previously proposed [31].

### CRISPR/Cas systems represent supplementary bacteriophage defense mechanisms for the species *L. monocytogenes*

Chromosomes of *L. monocytogenes* contain parts of a CRISPR/Cas-system implied in defense versus bacteriophages at three different loci (Additional file 7). These were identified by a combination of PILER-CR 1.02, CRT 1.1 and manual correction using BLASTN leading to slightly higher counts of repeat/spacer modules than previously published for strains 4a L99 and 1/2a EGD-e [21,68,69] (Additional file 8).

All strains bear a putative remnant of a CRISPR-system at ca. 0.5 Mb in strain 1/2a EGD-e which is not associated with any *cas* genes [37]. The distribution of spacers indicates, that ancestors of lineage I and II have lost the *cas* genes necessary to create new spacers inside this locus, leading to a relatively homogenous distribution, while strains of lineage III maintained this ability for a period long enough to completely differentiate their spacer sequences.

Locus 2 is located ca. 10kb adjacent to locus 1 and resembles the *Thermotoga neapolitana* (Tneap) subtype which consists of *cas6*, *cst1*, *cst2*, *cas5t*, *cas3* and *cas2*[70]. Homologues of this system exist in 4a L99, 7 SLCC2482 and 1/2b SLCC2755 at the same relative chromosomal position and no sequence remnants could be identified in other chromosomes, suggesting the insertion of this locus in a common ancestor of these strains. Spacers are identical in strains 7 SLCC2482 and 1/2b SLCC2755, while 4a L99 shows a completely different content.

Locus 3 is inserted into homologues of a lipoprotein gene (*lmo2595*) located at ~2.7 Mb relative to the chromosome of reference strain 1/2a EGD-e. It was found to be present in 1/2a SLCC5850, 7 SLCC2482, 1/2b SLCC2755 and 3b SLCC2540 without any local sequence homologies in other strains, implying insertion into a common ancestor of the former strains. This locus was found to contain *csn2*, *cas2*, *cas1* and *csn1* and thus classified as subtype *Neisseria meningitidis* (Nmeni)*.* Spacer content of locus 3 is clonal for strains 7 SLCC2482 and 1/2b SLCC2755 while 1/2a SLCC5850 and 3b SLCC3540 display mostly unique spacers, including a number of duplicates versus listeriaphages A500 and A118. Locus 3 belongs to subtype Nmeni which was previously described to rely on a *trans*-encoded sRNA (tracrRNA) located upstream of *csn1* and host factor RNase III in order to compensate for a missing endoribonuclease gene [30]. We could exclusively identify perfect matches of the 94 bp tracrRNA variant as expressed by *L. innocua* Clip11262 in all compared strains of *L. monocytogenes* bearing locus 3 at a position upstream of *csn1*. We thus hypothesize, that this locus functions according to the former principles and may only be able to silence foreign nucleic acids inside a host which is able to supply an RNase III enzyme.

All identifiable spacers (81/276) are directed versus known listeriaphages or related composite prophages. We also encountered multiple different spacers homologous to sequences of the same phage in the same array, as well as identical duplications of one spacer. It is tempting to speculate that inclusion of redundant spacer sequences increases the likelihood of a successful defense against the respective bacteriophage (ex.: A118, A500, B025). We never observed identical spacers to be present in multiple arrays, indicating a clear separation of all loci. No spacer was found to target chromosomal or plasmid sequences of species *L. monocytogenes* apart from integrated prophages, indicating that CRISPR/Cas does not serve further regulatory roles facilitated by direct base-pairing with target sequences [28,29].

In conclusion, we propose that an ancestor of genus *Listeria* contained a functional CRISPR locus 1 (*lmo0519-lmo0520*) that lost its associated *cas* genes during early evolutionary events. Interestingly, this locus was previously described as trans-acting small non-coding RNA RliB in strain 1/2a EGD-e indicated in control of virulence [35,37]. Thus, this remnant CRISPR array may have been adapted for regulation in 1/2a EGD-e and possibly other strains of the species. Five of 16 strains compared in this work still contain at least one of two types of putatively functional CRISPR/Cas systems indicating an ongoing selective pressure by bacteriophages. On the other hand, presence or lack of such a system does not correlate with number or type of prophages identified per strain and 11 strains neither bear a functional CRISPR/Cas system nor an increase of other defense mechanisms such as restriction modification systems (data not shown). We suggest that CRISPR/Cas represents an additional line of defense directed against bacteriophage attacks that can be gained by horizontal gene transfer and seems to be effective only for a subset of strains of genus *Listeria*. The variable nature of CRISPR-arrays suggests their future use in differentiating strains or lineages by typing procedures. Further research will now be necessary to determine the operational capability of locus 2 and 3 in the environment or host.

### Phylogenies compared – relationships between lineages, serogroups, serotypes and strains according to genomic and genetic content

This analysis used the complete genomic sequences of 19 strains of genus *Listeria* including those of related species being *L. innocua* 6a Clip11262, *L. welshimeri* 6b SLCC5334 and *L. seeligeri* 1/2b SLCC3954 to identify phylogenetic relationships.

**Figure 3:**
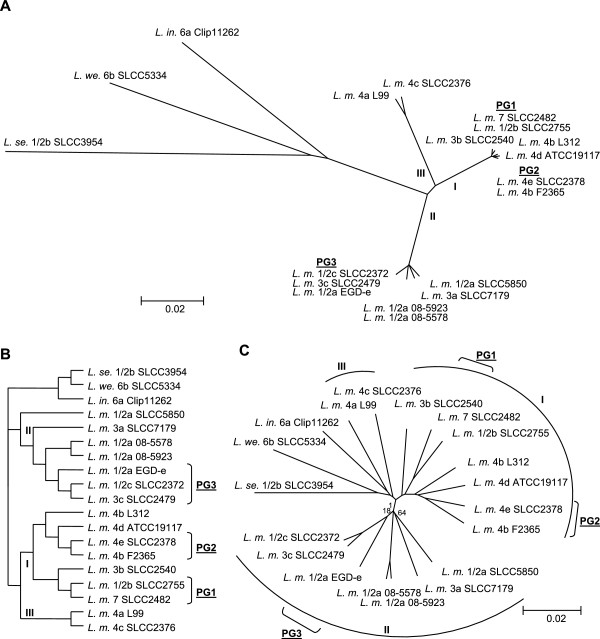
**Phylogenomic and -genetic trees. **(**A**) Neighbor joining tree based on an alignment of 2018 mutually conserved core genes (amino acid identity >60%, coverage >80%) of 19 strains of genus *Listeria*. Bootstrap support of 100 replicates was always found to be above 80% and thus omitted. Lineages of *L. monocytogenes* are marked in roman letters and phylogenomic groups (PG) describe closely related strains. (**B**) Data of panel A transformed to as cladogram to highlight branching. **(C**) Neighbor joining tree of gene content (presence/absence). Only bootstrap support values below 80% (100 replicates) are indicated.

In order to enable phylogenetic clustering we created a well-supported (bootstrap >80%) core-genome tree based on an alignment of all concatenated core genes (2018) of 19 strains using Mugsy [71] (Figure 3A/B). This tree shows distances between strains based on small adaptations inside mutually conserved genes, which translate into an approximate timeline when assuming consistent rates of evolution. We found that strains of species *L. monocytogenes* clustered inside three clearly separated lineages in support of previous observations [7,8]. Lineage III contains serotypes 4a and 4c, lineage II includes 1/2a, 1/2c, 3a and 3c and lineage I bears strains of serotypes 1/2b, 3b, 4b, 4d, 4e and 7. Differentiation leading to separate serotypes apparently had little impact on the placement of branches apart from the general lineage. We identified the closest relationships between strains of different serotypes being 1/2b SLCC2755, 7 SLCC2482 (termed phylogenomic group 1 or PG1) and 4e SLCC2378, 4b F2365 (PG2) in lineage I, as well as 1/2a EGD-e, 1/2c SLCC2372, 3c SLCC2479 (PG3) in lineage II, with the exception of clonal strains 08–5578 and 08–5923 which both belong to serotype 1/2a. There is a clear correlation of PGs with previously determined CCs, whereby PG1 strains were classified as CC3, PG2 strains as CC1, and PG3 strains as CC9 [7]. Strains of serotypes 4e and 4d were found on a branch displaying strain 4b L312 as its oldest ancestor in support of a previous hypothesis indicating serotype 4b as ancestral state for serotypes 4e and 4d [7].

We additionally clustered all strains based on the accessory gene content (presence/absence of 2953 genes) to identify the impact of gene-scale indels, which includes most horizontal gene transfer events [72] (Figure 3C). This methodology was shown to be biased towards a tree topology that parallels convergence in lifestyle and thus displays a phenotypical relationship among the compared strains [73]. The resulting tree was found to be well supported (>80% bootstrap) with the exception of the placement of branches neighboring the central *L. monocytogenes* junction, implying early indels and recombination that lead to inconsistent topologies.

If only gene gain and loss are taken into account, lineages of *L. monocytogenes* are closely related to other listerial species, indicating that large evolutionary timeframes shown by the SNP-based core-genome tree resulted in a low number of conserved gene-scale indels.

The opposite is apparent when considering phylogenomic groups, which were found to be closely related in the core-genome tree but to a much lesser degree considering gene content, implying a number of young indels. Interestingly, phylogenomic groups are located at the end of shorter common branches in the gene content tree, which is due to a small number of exclusively conserved genes (PG1: 28, PG2: 20, PG3: 22, primarily hypothetical and truncated genes) (Additional file 1). Thus, strains of phylogenomic groups can be considered closely related but do not necessarily share the same niche or phenotype. Other branches are supported by a varying number of conserved and predominantly hypothetical genes (ex. 4b L312, 4b F2365, 4d ATCC19117, 4e SLCC2378: 18 genes; 3b SLCC2540, 1/2b SLCC2755, 7 SLCC2482: 5 genes) that are distributed along the chromosomes in small modules.

We identified three topological changes between core-genome and gene content tree hinting at shared indels that run contrary to the phylogenomic signal of core-genome SNPs. Removal of genes related to mobile genetic elements (34% of accessory genes) from the gene content matrix resulted in a topology very similar to the core-genome tree. Thus, large-scale insertions, which resulted mainly from bacteriophage integration, run contrary to the “true” phylogenetic signal by inserting many genes in one event as well as by putative parallel insertions into different strains. The only remaining difference was observed considering a common branch for strains of lineage III and apathogenic species, highlighting small-scale indels as causative force. This supports a previous hypothesis suggesting lineage III as a possible deleterious intermediate state between lineages I/II and apathogenic species [7,9,21,74,75].

Interestingly, the majority of accessory genes of species *L. monocytogenes* were either scattered along the chromosomes (46%) or found inside hyper variable regions (20% when excluding MGE) and thus likely originated from a wide range of diversifying forces. Gradual change seems to be a superior factor for the evolution of gene content of *Listeriae* when compared to large-scale insertions of multiple genes by mobile elements.

In summary, tree topologies based on a core-genome alignment and gene content were found to be highly similar despite the obfuscating influence of mobile genetic elements. Other studies on *Rickettsia/Orienta* species and *E.coli/Shigella* found considerable differences in the respective phylogenies indicating more distinct evolutionary histories for the gene repertoires involved [44,76]. The relative correspondence of SNPs and gene-scale indels in genus *Listeria* could be a result of differential acquisition and loss of genes in accordance to various evolutionary descents as previously described considering other genera [77,78].

### Frequent loss and disruption of known virulence-associated genes may explain observed phenotypic attenuations

**Figure 4:**
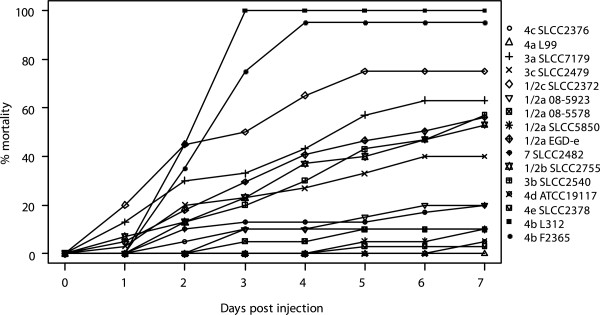
***Galleria mellonella *mortality rates.** Mortality rates of *Galleria mellonella* larvae over the course of seven days post injection. Respective standard deviations can be found in the supplementary material (Additional file 10).

About one third of the genes which displayed compelling evidence for involvement in the infectious process were found to be absent or to code for a truncated protein in at least one of the strains studied, putatively impacting the disease phenotype (Additional file 9) [18,19,79-84]. Rates of mortality of larvae in the *Galleria mellonella* model system indicative of pathogenicity showed that strains of serotype 4b killed most larvae, followed by 1/2c SLCC2372, 3a SLCC7179, 1/2a EGD-e, 1/2b SLCC2755, 3b SLCC2540 and 3c SLCC2479 (Figure 4, Additional file 10). The remaining strains displayed a low degree of pathogenicity in this model, which was described to emulate many aspects of *Listeria* infection seen in vertebrates [85]. Nonetheless, limits of the insect model in forecasting effective human infection become obvious regarding human listeriosis outbreak strains 1/2a 08-5923/08-5578, which only lead to low rates of host mortality following *Galleria* infection. Approximately half of the strains compared in this study were furthermore found to be virulence attenuated as assessed by low invasion rates of epithelial cells [50]. *Galleria* mortality and HeLa cell invasion rates correlated for 6 strains (1/2b SLCC2755, 4b L312, 4c SLCC2376, 7 SLCC2482, 4e SLCC2378, 4d ATCC19117), while 4 strains killed the majority of larvae without being able to invade HeLa cells (3a SLCC7179, 3c SLCC2479, 1/2c SLCC2372, 3b SLCC2540). The latter observation indicates that the respective strains are able to invade other cell types in order to infect an invertebrate host. In order to assess maximum growth rates in a rich medium, the compared strains were furthermore grown in BHI medium at 37°C (Additional file 11). The only outlier was found to be strain 1/2a SLCC5850, which grew considerably slower than the other strains.

In order to correlate phenotypes with genomic differences we performed detailed analyses of virulence-associated genes that allow us to present hypotheses on the evolutionary descent of these changes (Additional file 12). In short, deletions affecting primary virulence genes *prfA* (1/2a SLCC5850), *plcA* (3a SLCC7179), *inlA* (3c SLCC2479), and *inlB* (4b F2365) were identified in four strains [32,86]. A number of surface-associated genes were found to be absent from strains of lineage III and especially from strain 4a L99 [7-9,21,75]. Further deletions which putatively interfere with regulation of the SigB regulon during stress are related to genes *rsbS* (1/2c SLCC2372), *rsbV* (4d ATCC19117) and *rsbU* (3c SLCC2379) [87-89]. The BHI growth attenuation of 1/2a SLCC5850 may result from the specific absence of 12 genes found in all other compared strains, coding for various proteins involved in energy production/conversion and metabolism (Additional file 1).

In conclusion, strains of *L. monocytogenes* frequently lose determinants of pathogenicity leading to virulence-attenuated phenotypes, which may be advantageous in some environments, especially considering lineage III [7-9,21,75]. Interestingly, highly invasive and/or pathogenic strains of serotypes 4b, 1/2a, 1/2b, and 1/2c also displayed a range of deletions here, indicating a certain amount of redundancy of these functions [18,31,32].

### Distribution of surface-associated genes displays conserved lineage-backbones with strain-specific adaptations

A detailed examination was undertaken to spot relevant patterns of presence or absence of surface-associated genes mediating interaction with the environment and the infected host, and to invoke evolutionary explanations (Additional file 13, Additional file 14).

To conclude, genes bearing P60 or LysM domains showed little variation among the strains studied (Additional file 15) [22,23]. Between 6 and 16 non-core lipoprotein coding genes were identified, indicating some differentiation. These were frequently located in chromosomal hotspots of horizontal gene transfer and found inside or adjacent to prophage insertions, hinting at putative methods of transmission. Interestingly, all strains of epidemic lineage I show an exclusive gene (*LMOf2365_1974*) with both LPXTG and GW domains, which may become a future research target when considering the role of cell wall anchored modulators of virulence or pathogenicity.

Internalins are involved in cell adhesion and invasion of host cells and contain a leucine-rich repeat (LRR) domain indicated in protein-protein interaction (Additional file 16) [24-26]. InlB B-repeats represent a hallmark of previously described virulence-associated internalins [90], and were identified in 15 clusters, thus increasing the probability of the respective genes to be involved in host-pathogen interaction. The distribution of putative internalins revealed that only four of 42 homology clusters are mutually conserved, confirming previous observations of diversity, especially considering lineages II and III [91,92]. A number of known virulence-associated internalins were absent in a subset of strains, putatively resulting in a reduced number of infectable cell types (lineage III: *inlC* and *inlF*, 4a L99: *inlGHE*, *inlI* and *inlJ*, 3c SLCC2479: *inlA*, 4b F2365: *inlB*) [9,21,32,75,92]. The absence of *inlC* in strains of lineage III may have been caused by a deleterious transposition moving two adjacent lipoprotein coding genes (*lmo1264-5*) by approximately 600kb to replace the internalin (Additional file 17). Interestingly, we identified different versions of *inlF* and *inlJ* in lineage I as compared to lineages II/III, putatively resulting in different adhesion properties and implicated in host tropism [93]. Only one internalin was found to be specific and mutually conserved for lineage I (*LMOf2365_0805*), indicating this gene for further research regarding virulence.

Taken together, we found that most surface-associated genes are either mutually conserved or were likely present in an early ancestor of a lineage, implying a fixed core-functionality that is rarely complemented by strain-specific additions confirming previous observations [22-24]. Nonetheless, we identified a number of novel surface-associated genes, including their distribution among all serotypes of species *L. monocytogenes*, thereby presenting a pool of candidates for future analysis considering virulence and pathogenicity.

### Ancestral genes of serotypes, serogroups and lineages reveal new marker and virulence-associated genes while strain-specific genes rarely represent an obvious extension of functionality

In order to identify conserved ancestral genes which may be important for the differentiation of lineages, we collected genes that were found in all strains of a lineage (>60% amino acid identity, >80% coverage) and absent in all strains of other lineages (Additional file 18). Thus, 33 lineage-III-specific, 22 lineage-II-specific and 14 lineage-I-specific core genes could be identified, which are largely supported by previous microarray-based studies [9,47]. Due to analyses of genetic localization and sequence composition, we want to propose the hypothesis that ancestral strains of lineage I and III diverged from lineage II by loss of genes related to carbohydrate metabolism and gain of hypothetical and surface-associated genes. This theory is based on the following observations: (1) distinct lineage core genes of lineage II predominantly include PTS systems and ABC transporters involved in carbohydrate metabolism organized in three operon-like islands, while those of lineages I and III mainly consist of scattered hypothetical and surface-associated proteins, (2) specific core genes of lineage II display no deviation from the average G/C content of the respective chromosome or codon usage disparities frequently associated with horizontal gene transfer, (3) strains of lineages I and III contain putative sequence remnants of some of these genes (*lmo0734, lmo1060*, ~60bp with >75% nucleotide identity), (4) neighborhood and sequence of specific core genes of lineages I and III show more ambiguous patterns including putative insertions, especially considering surface-related proteins (data not shown). According to this hypothesis, ancestral strains of lineages I and III have lost genes related to carbohydrate metabolism and instead gained genes coding for surface-associated proteins serving different needs considering nutrients and interaction with the environment.

The adaptation of strains of lineage III furthermore included loss of genes implicated in food preservation measures, pathogenicity, or virulence as previously described [9,10,21,48,94,95]. We identified 45 genes found to be conserved in 13 out of 14 strains of predominantly human listeriosis-related lineages I and II while being absent from both strains of lineage III (Additional file 19). These comprise genes coding for 16 hypothetical proteins, 14 metabolic enzymes, 6 surface-associated proteins and 4 transcriptional regulators. Affected metabolic pathways include non-mevalonate isoprenoid, fructose and arginine biosynthesis, as well as a nitroreductase and a hydrolase [9,10,21,48,94,95]. Other genes related to stress resistance exclusively conserved in these lineages include the intracellularly up-regulated A118-like prophage rest also known as monocin or *lma*-operon [21]. Furthermore, lineage III does not contain genes coding for multiple internalins and amidases associated with invasion (*inlF, inlC*, *lmo0129, lmo0849*) [9,21,47]. In summary, strains of less virulent and less pathogenic lineage III mainly differ from the other two lineages by loss of genes involved in metabolism, stress resistance and surface-associated functions implied in adaptation to the complex inter- and intracellular environment inside the host, as well as resistance towards food preservation measures [9,21,47].

We also tried to identify exclusive indels for serogroups or –types that are represented by at least two strains in this analysis in order to uncover ancestral sequences (Additional file 20). We found nine genes to be specific for all strains of serogroup 4, while 16 genes are specifically absent, most of which were already described to be responsible for differences in teichoic acid composition [32,95]. Neither strains of serogroups 3 or 1/2, nor of serotypes 1/2a or 4b show exclusive gene indels, indicating that the respective variable antigens either result from minor changes inside coding genes, from differences located in intergenic regions (ex. promoters, imperfect automatic prediction of ORFs, operon structures, etc.) or from heterogeneous causes.

In order to assess the impact of recent adaptations, strain-specific genes were examined (Additional file 21). Between 11 (3c SLCC2479) and 177 (4a L99) genes per strain were classified as specific, including 0 (4b L312) to 93 (4a L99) genes inserted by a set of previously determined mobile genetic elements dominated by specific prophages. Up to 37 strain-specific genes were found to be fragments of genes either split or truncated by the insertion of a premature stop-codon (“pseudogenes”). Most of these are transporters, metabolic enzymes or regulators and in many cases associated with virulence or pathogenicity as described previously [8]. Strains 1/2a SLCC5850 and 7 SLCC2482 displayed an overrepresentation of fragmentary CDS, which may mark the recent onset of a reductive adaptation. Strain 4b L312 was isolated from cheese and shows a specific insertion of an additional lactose/cellobiose PTS (*LMOL312_2315-20*), which could represent an adaptation to dairy products. A specific element found in strain 3b SLCC2540 resembles the bacteriocin transport and resistance system lantibiotic sublancin 168 (*LMOSLCC2540_2733-40*, up to 28% amino acid identity at 100% coverage) [96]. We found no homologue to the *sun*A bacteriocin peptide, suggesting either export of a different bacteriocin or an exclusive function in resistance to these molecules. Interestingly, eight non-homologous restriction-modification systems were also found to be strain-specific, confirming observations of their “selfish” and competitive nature [97].

### Small non-coding RNA candidates of *L. monocytogenes* are largely conserved within the species

Previous transcriptomic analyses uncovered 210 regulatory sRNA candidates expressed in *L. monocytogenes,* some of which have been implicated in adaptation to iron limitation, oxidative stress, low temperature or intracellular survival [35-41]. We identified homologues of these in all compared strains in order to identify patterns associated with evolutionary descent and possible involvement in the infectious process using sRNAdb [98] (Additional file 22).

Only 43 of these were found to be accessory sRNAs, defined as being absent from at least one compared strain, including 20 sRNAs that are only present in a subset of strains of lineage II. Approximately half of those differentially distributed sRNAs, that were previously suggested to be involved in virulence or pathogenicity by growth attenuation of deletion mutants in mice (*rli33-1, rli38, rli50*) or by intracellular up-regulation in macrophages (*rli24, rli28, rli29, rliC, rli85, rli95, rli48, rli98, rliG*) were also exclusively present in a varying subset of strains of lineage II [35,36]. It should be noted that this subset never included strain 3a SLCC7179, implying that ancestral strains of 3c SLCC2479, 1/2c SLCC2372 and serotype 1/2a contained a specific range of sRNAs in order to adapt to the environment and to modulate the infectious process.

We found only *rli38, rli62,* and *rliG* to be specifically present in strain 1/2a EGD-e, whereby the latter two sRNAs inserted as part of specific prophage A118 (MGE-13). Transcriptional activation of prophage genes was reported previously, but an impact on phenotype due to prophage-related sRNAs has still to be elucidated in species *L. monocytogenes*[21,50].

Interestingly, strain 3a SLCC7179 shows a fragmented homologue of *ssrA* (*tmRNA*, 391/500 bp = 78% coverage) necessary for the *trans*-translation of mRNAs that lack a natural stop-codon. Some strains of *E. coli* contain an alternative sRNA termed *afrA* (*yhdL*), which can serve as a possible replacement but was found to be absent from all compared strains [99]. Thus, we speculate that either the shortened *ssrA* gene is still functional or that species *L. monocytogenes* or specifically strain 3a SLCC7179 harbor another yet unknown system to recycle stalled ribosomes and incomplete polypeptides.

In summary, evolution of small non-coding RNAs represents an ongoing process in species *L. monocytogenes.* This excludes all riboswitches found to be mutually conserved in all compared chromosomes, strengthening a hypothesis implicating *cis* acting RNA regulation as an ancient mechanism [36]. Small non-coding RNA transcriptomic analysis of strains of lineages I and III will now be required to uncover their specific regulatory networks on this level.

### LisDB – a comparative genomics server for the *Listeria* research community

A large part of the analysis presented in this study is based on the GECO comparative genomics software [53]. We have created a public web-server that includes all published chromosomes and plasmids of genus *Listeria,* as well as a subset of genomes of related genera. The main function of this tool is the identification of homologous genes between replicons to uncover relationships of genomic regions or complete pan-genomic distributions. These data can be visualized graphically or exported in the form of tab-delimited lists. Among the latter are matrices sorted for conservation in selected replicons or for synteny according to a reference strain. Gene gain and loss between two replicons can be identified and nucleotide or amino acid sequences can be exported. GECO-LisDB is accessible at the following address: http://bioinfo.mikrobio.med.uni-giessen.de/geco2lisdb.

## Conclusions

*Listeria monocytogenes* represents a well-characterized pathogen and model system for infection research. Extension of fully sequenced genomes by 11 strains to include all serotypes of the species allowed evolutionary analyses of unprecedented depth. Comparative examination in conjunction with public data revealed that (i) the species pan-genome is highly stable but not closed, (ii) accessory genes are mainly located in defined chromosomal regions (nine hyper variable hotspots, nine different prophages, three transposons, and two mobilizable islands) constituting primary loci of gene-scale species evolution, (iii) potentially functional CRISPR/Cas systems of different subtypes are infrequent but may shape genome diversity, (iv) evolutionary distances observed between lineages of *L. monocytogenes* and apathogenic species are mostly the result of SNPs rather than gene-scale indels that are rarely commonly inherited, highlighting the potential impact of small-scale mutation on long-term development, (v) frequent loss or truncation of genes described to be vital for virulence or pathogenicity was confirmed as a recurring pattern, especially for lineages II and III.

The presence or absence of genes among all serotypes of species *L. monocytogenes* uncovered by this study will be helpful for further diagnostic, phylogenetic and functional research, and is assisted by the comparative genomic GECO-LisDB analysis server (http://bioinfo.mikrobio.med.uni-giessen.de/geco2lisdb).

## Methods

### Sequencing

The 11 isolates to be sequenced were selected to achieve full coverage of serotypes of species *L. monocytogenes* as previously characterized by MLST, PFGE, and MALDI-TOF [7,100] (Table 1). DNA was purified per strain using Epicentre’s MasterPure gram-positive DNA purification kit as recommended by the manufacturer and ten μg of genomic DNA were used for library-preparation following the manufacturer`s constructions (Roche 454 Life Science GS FLX Shotgun DNA Library manual). Sequencing was performed on a 454 GS-FLX system using GS FLX Standard Chemistry. Between 213437 and 297585 reads per strain were *de novo* assembled with the GS Assembler (Newbler 1.1.03.24). The resulting contigs were compared to published strains of *L. monocytogenes* covering major lineages (eg. 4a L99, 1/2a EGD-e, 4b F2365) using Mauve for scaffolding purposes. Differing layouts were assessed manually and joined to a preliminary consensus order. PCR-based techniques followed to close the remaining gaps partially assisted by Minimap (unpublished software) to identify specific primer pairs. This software combines BLASTN and Primer3 in order to identify primer candidates located at the edge of each contig. Primer candidates were selected to not target repetitious sequences (>70% nucleotide identity at >50% coverage). PCRs were sequenced with Sanger ABI Big Dye technology (Applied Biosystems). Sanger reads were incorporated into the assembly using the GAP4 software package v4.11 and SeqMan (Lasergene 5). A total of 487 gaps were closed this way resulting in finished sequences covered from either high-quality 454-reads or Sanger-reads. The completed chromosomes achieved mean coverages between 16-26x and 99.67–99.93% of the bases carried Q40 or higher quality scores. The final gap in the chromosome of *L. monocytogenes* 7 SLCC2482 was marked with a sequence of 100 Ns. Sequencing and finishing procedures were carried out by the Goettingen Genomics Laboratory (Goettingen, Germany), the Institute of Medical Microbiology of the Justus-Liebig University (Giessen, Germany), Roche (Germany), and Agowa (Berlin, Germany). All replicons were deposited in the EMBL database (see Table 1 for accession numbers).

### Annotation

Automatic annotation was performed by GenDB, which includes steps for the identification of protein coding sequences (CDS), rRNA and tRNA genes as well as similarity searches against major gene and protein databases [101]. The annotation was enriched using a separate bidirectional best BLASTP step (>80% amino acid identity, >90% coverage) to incorporate data from *L. monocytogenes* 4a L99 (EMBL-Bank: FM211688) and the surface protein prediction software Augur using default parameters [102]. Further annotation was extracted from publications dealing with specific classes of genes such as CRISPR/Cas [70] and known internalins [103]. All information obtained was joined and mapped onto a list of clusters bearing all genes of eleven strains (homology >80% amino acid identity, >90% coverage) using GECO [53] and manually curated according to the following rules with decreasing relevancy: (1) homology to a known gene group (e.g. Cas, internalin, surface-associated) (2) homology to a coding sequence from strain 4a L99, (3) classification as a surface-associated protein-coding gene according to Augur, (4) at least partial homology (>60% amino acid identity, >80% coverage) to a gene family found in Pfam [104] (5) or at least partial homology (>60% amino acid identity, >80% coverage) to a gene found in the NCBI nr database. A manual scan of the complete chromosomes using the GECO visualization interface revealed a number of genes that were fragmented (at least 25% shorter than orthologous genes of reference strains 4a L99, 1/2a EGD-e, and 4b F2365) due to the presence of premature stop-codons and thus annotated as putative fragmentary genes. All automatic annotations were adapted in order to achieve congruent annotations for modules of genes. If no annotation was possible according to these rules, the respective putative protein-coding gene was labeled as a hypothetical protein.

### Comparative analyses

Homologous coding sequences were identified by BLASTCLUST [105] as implemented in the comparative genomics software GECO [53]. The standard similarity criterion was set to a minimum of 60% amino acid identity and 80% coverage of both proteins. Chromosomal regions were checked manually using the comparative genome browser of GECO in order to find orthologous CDS which satisfied the homology criteria and were located in a syntenic region in comparison to a reference strain. In some cases a stricter analysis based on 80% amino acid identity and 90% coverage was additionally employed to reduce the number of false positives. In order to avoid excessive redundancy, we denote only one gene of a homologous cluster in brackets, which can be further assessed using either the GECO LisDB server (http://bioinfo.mikrobio.med.uni-giessen.de/geco2lisdb) or the supplementary homology matrix (Additional file 1).

### Pan-genome analysis

The pan-genome size of *L. monocytogenes* was predicted based on the chromosomes of 16 sequenced strains compared in this study. We employed the standard BLASTCLUST homology cutoff of 60% amino acid identity and 80% coverage for this analysis. Chromosomes were added 10000 times in a randomized order without replacement, and the number of core (mutually conserved), and accessory (found in at least one but not all strains) genes was noted using GECO. Since mean and median values for each step showed little variation, mean numbers of gene classes were plotted. In order to predict a possible future pan-genomic distribution for this species we performed a power law fitting as described previously [55].

### Identification of large insertions

The colinearity of chromosomes of *L. monocytogenes* allowed a relatively simple method to identify large insertions. First we masked the sequence inversion surrounding the oriC in strain 08–5923 (LM5923_2737-0270) and 08–5578 (LM5578_2788-0270) by reordering coding sequences to follow the usual chromosomal layout as found in strain 1/2a EGD-e. CDS were then compared in a bidirectional best BLASTP analysis using similarity criteria of more than 60% amino acid identity and 80% coverage of both CDS. Core-CDS existing in all compared strains were identified by single linkage clustering (AB + BC = ABC). All core-CDS showing a break in the synteny (translocation, inversion) relative to reference strain 1/2a EGD-e were removed from the pool. Finally, the number of CDS located between syntenic core-CDS was plotted as a bar chart per strain. Exact borders of mobile genetic elements were identified based on annotation, deviation of GC-content and comparative analysis with sequenced phages and strains of genus *Listeria*.

### CRISPR/Cas analysis

Spacer/repeat-arrays were identified with PILER-CR 1.02 and CRT 1.1 using standard parameters with the exception of maximum repeat length, which was increased to 40 [68,69]. Resulting arrays were combined and controlled manually leading to the removal of eleven false positives inside LRR- and LPXTG-domain containing coding sequences. Consensus sequences of repeats of remaining loci were employed for a BLASTN search versus chromosomes of all strains resulting in the identification of multiple decaying spacer/repeat modules that had been ignored by Piler and CRT due to repeat sequence mismatches of up to 20%. Spacers were compared to 10 published bacteriophages of genus *Listeria* (A006: NC_009815, A118: NC_003216, A500: NC_003216, A511: NC_009811, B025: NC_009812, B054: NC_009813, P100: NC_009813, P35: NC_009814, P40: EU855793, PSA: NC_003291), 16 chromosomes and 4 plasmids of strains of this study and the NCBI nt-database using BLASTN. Alignments showing up to 1 mismatch were deemed homologous. Finally, all spacers where compared to each other using BLASTCLUST considering perfect matches only and mapped to mirror the order of spacers inside the respective loci to visualize the degree of relatedness (Additional file 3, software BlastclustToMatrix available upon request). Softening of the homology cutoffs to 80% nucleotide identity at 80% coverage did not result in a meaningful increase of matches. *cas* genes were identified by sequence homology to published data found in the NCBI NT database and Pfam [104].

### Phylogeny

A phylogenetic core-genome tree was created based on mutually conserved core CDS of all compared strains including out-group strains *L. innocua* 6a Clip11262, *L. welshimeri* 6b SLCC5334 and *L. seeligeri* 1/2b SLCC3954. These were extracted from a GECO homology matrix (amino acid identity >60%, coverage > 80%) (Additional file 1) following removal of all clusters showing paralogues. A total of 2018 protein coding genes were concatenated resulting in approximately 2 Mb of nucleotide sequence information per strain. The data was aligned using Mugsy [71] and resulting locally collinear blocks were joined per strain and imported into MEGA5 and SplitsTree4 [106,107]. Based on the alignment we created multiple phylogenomic trees (maximum parsimony, minimum evolution, neighbor joining) including 100 bootstrap replicates. Since tree topology was identical in all cases and relative branch lengths showed little variation, we only present trees based on the neighbor joining algorithm.

In order to identify the impact of indels on phylogeny we built a second tree based on the presence and absence of 2953 accessory genes using GeneContent [72]. Distance between strains was calculated with the Jaccard coefficient [108] and a tree was inferred using the neighbor joining reconstruction method including 100 bootstrap replicates.

### Identification of surface-associated genes and putative internalins

Surface-associated genes were identified based on sequence similarity to known motifs (P60, LysM, GW, LRR, LPXTG, lipo) using various Hidden Markov Models (HMM) and SignalP as implemented by Augur [102]. A domain was considered present if HMM e-value < 10 and HMM score > 5. All surface-associated homology matrices were created using a higher standard cutoff (80% amino acid identity, 90% coverage) in order to achieve a higher degree of resolution and thus identify even small amounts of sequence dissimilarity. Clusters showing paralogous CDS were manually split according to a GECO synteny analysis.

All CDS containing a leucine rich repeat (LRR) domain were assumed to be putative internalins and checked for the presence of a signal peptide. False positives and negatives as revealed by synteny analysis were corrected manually and the homology cutoff was reduced to 50% identity and 40% coverage if necessary. Apprehension of internalin-types based on predicted internalins from a previous study [103] as well as domains identified by Augur completed the analysis.

### Measurement of bacterial growth

Bacterial cultures were grown over night at 37°C in brain heart infusion broth (BHI) and diluted 1:200 the next day for fresh cultures. Automated measuring at 37°C was performed using the Infinite 200 plate reader (Tecan) in 96-well plates with 150 μl volume/well.

### *Galleria mellonella* infection model

In order to assess the degree of pathogenicity of the 16 strains studied, the insect model *Galleria mellonella* was employed [85]. While this model is unable to mimic all features of vertebrate hosts, a number of listerial virulence genes are generally needed for infection in mammals as well as in invertebrates. In short, bacteria were serially diluted using 0.9% NaCl to a concentration of 10^8^ cells/ml. The dilution was plated out on BHI agar plates to calculate the inoculum injected. Ten μl (10^6^ bacteria) inoculum were injected dorsolaterally into the hemocoel of last instar larvae using 1 ml disposable syringes and 0.4 × 20mm needles mounted on a microapplicator as described previously. After injection, larvae were incubated at 37°C. Larvae were considered dead when they showed no movement in response to touch. No mortality of *Galleria* larvae were recorded when injected with 0.9% NaCl. Two different versions of these independent experiments were conducted. Strains 1/2a 08–5923, 1/2a 08–5578, 1/2a SLCC5850 and 4b F2365 were injected into 10 animals each and the experiment was performed 2 times per strain. The remaining strains were injected into 20 animals each including 3 repetitions. Mean percental mortality rates of 2 × 10 and 3 × 20 larvae were noted, respectively.

### Analyses of sRNAs

Multiple studies have previously determined small non-coding RNA candidates of species *L. monocytogenes* that were classified as intergenic sRNAs, antisense sRNAs, or cis-regulatory RNAs (including riboswitches) [35-41]. A consensus list was created, whereby candidate sRNAs overlapping by at least 50% were merged to one putative long transcript. Homologues of these 210 sRNA candidates were identified in all compared strains using a minimum BLASTN cutoff of 60% nucleotide identity and 80% coverage as applied by the sRNAdb software [98] (Additional file 22).

## Competing interests

The authors declare that they have no competing interests.

## Authors’ contributions

CK carried out bioinformatic tasks related to the sequencing process, performed annotation and comparative analyses and drafted the manuscript. AB participated in the annotation and bioinformatic analyses and assisted in drafting the manuscript. MM performed phenotypic experiments. AS performed the genomic sequencing. AG participated in the design of the study. RD participated in the design of the study. SB assisted in drafting the manuscript. TH conceived of the study, participated in its design and coordination and assisted in drafting the manuscript. TC participated in the design of the study and assisted in drafting the manuscript. All authors read and approved the final manuscript.

## Supplementary Material

Additional file 1 **Species homology matrices.** General homology matrices showing the distribution of all coding sequences among 16 strains of species *L. monocytogenes* and 19 strains of genus *Listeria* at different cutoffs. This table is sorted for maximum conservation (core genes = top, specific genes = bottom). Click here for a file

Additional file 2 **Insertional hotspot ranges.** Hotspots showing at least three separate insertions denoted by locustag ranges. Click here for a file

Additional file 3 **Comparative genomic GECO figures of hyper variable hotspots.** Comparative GECO depictions of insertional hotspots highlighting extensive mosaicism. Click here for file

Additional file 4 **Mobile genetic elements.** Distribution of mobile genetic elements ordered by relative position in the chromosome of *L. monocytogenes* 1/2a EGD-e. Click here for a file

Additional file 5 **Comparative genomic GECO figures of transposons ICELm1 and TN554.** Comparative GECO depiction using a homology measure of 60% amino acid identity and 80% coverage. Displays content and conservation of two transposons. Click here for file

Additional file 6 **Comparative genomic GECO figures of IS3 elements.** Comparative GECO depiction using a homology measure of 60% amino acid identity and 80% coverage. Displays duplication of IS3-like transposon. Click here for file

Additional file 7 **Comparative genomic GECO figure of CRISPR/Cas loci.** Comparative GECO depictions of three CRISPR/Cas loci using a minimum CDS homology measure of 60% amino acid identity and 80% coverage.Cas genes and spacer/repeat arrays are framed. Locus 1 displayed no associated Cas genes. Locus 3 includes a *trans*-acting sRNA called tracrRNA that was described to compensate for a missing endoribonuclease in conjunction with host factor RNase III. Click here for file

Additional file 8 **CRISPR/Cas loci.** Homology matrices and positions of CRISPR/Cas genes and associated arrays of three loci. Spacers were additionally mapped versus the NCBI nt database to identify possible target sequences. Click here for a file

Additional file 9 **Known virulence genes.** Homology matrix of known virulence genes. Click here for a file

Additional file 10 ***Galleria *standard deviations.** Standard deviations calculated for independent experiments considering mortality rates of *Galleria mellonella* larvae over the course of seven days post infection. Click here for a file

Additional file 11 **Growth curves BHI.** Growth of *L. monocytogenes* in BHI medium at 37°C. Click here for file

Additional file 12 **Detailed analyses of reductive evolution of virulence-associated genes.** In-depth information about previously described virulence and pathogenicity indicated genes that are absent or truncated in one of the compared strains. Click here for file

Additional file 13 **Plot of Surface-associated CDS.** Bar plot depicting the distribution of all surface-associated protein coding genes among studied strains. Click here for file

Additional file 14 **Distribution of surface-associated genes displays conserved lineage-backbones with strain-specific adaptations.** Detailed analysis of presence and absence of surface-associated genes. Click here for file

Additional file 15 **Surface-associated CDS.** Homology matrices of genes containing a surface-associated domain (NLPC/p60, LysM, GW, LRR, LPxTG, Lipobox, signal peptide). Click here for a file

Additional file 16 **Internalins.** Homology matrix of genes containing a leucine rich repeat domain and an optional signal peptide. Click here for a file

Additional file 17 **Putative transposition of lipoproteins *lmo1264-5 *in lineage III.** Comparative GECO depiction using a homology measure of 80% amino acid identity and 90% coverage. Displays the putative transposition of lipoproteins *lmo1264-5* in lineage III into the locus that putatively held *inlC* previously. Click here for file

Additional file 18 **Lineage-specific CDS.** Homology matrix of coding genes specifically present in one lineage. Click here for a file

Additional file 19 **Lineage I/II exclusive CDS.** Homology matrix of genes conserved in 13/14 strains of lineages I and II, while being absent from both strains of lineage III. Click here for a file

Additional file 20 **Serogroup and –type ancestral indels.** Homology matrix of CDS found to be commonly present or absent (ancestral indel) for either one or multiple serogroups or -types. Click here for a file

Additional file 21 **Strain-specific CDS.** Homology matrix of coding genes specifically present in one strain. Click here for a file

Additional file 22 **Small non-coding regulatory RNAs.** Homology matrix of sRNA candidates. Click here for a file
