# Supplementary material for: Reassessment of the Listeria monocytogenes pan-genome reveals dynamic integration hotspots and mobile genetic elements as major components of the accessory genome
Source: BMC Genomics. 2013 Jan 22;14:47. doi: 10.1186/1471-2164-14-47 (PMC3556495; doi:10.1186/1471-2164-14-47)

Hypervariable hotspot 1 (lmo0061-lmo0075)

Based on a homology cutoff >60% amino acid identity and >80% coverage. Black arrows indicate mutually conserved core genes. A black border denotes a deviation from the average codon usage of the chromosome. Loci of strains 1/2a 08-5923 and 08-5578 were reversed to enhance clarity.

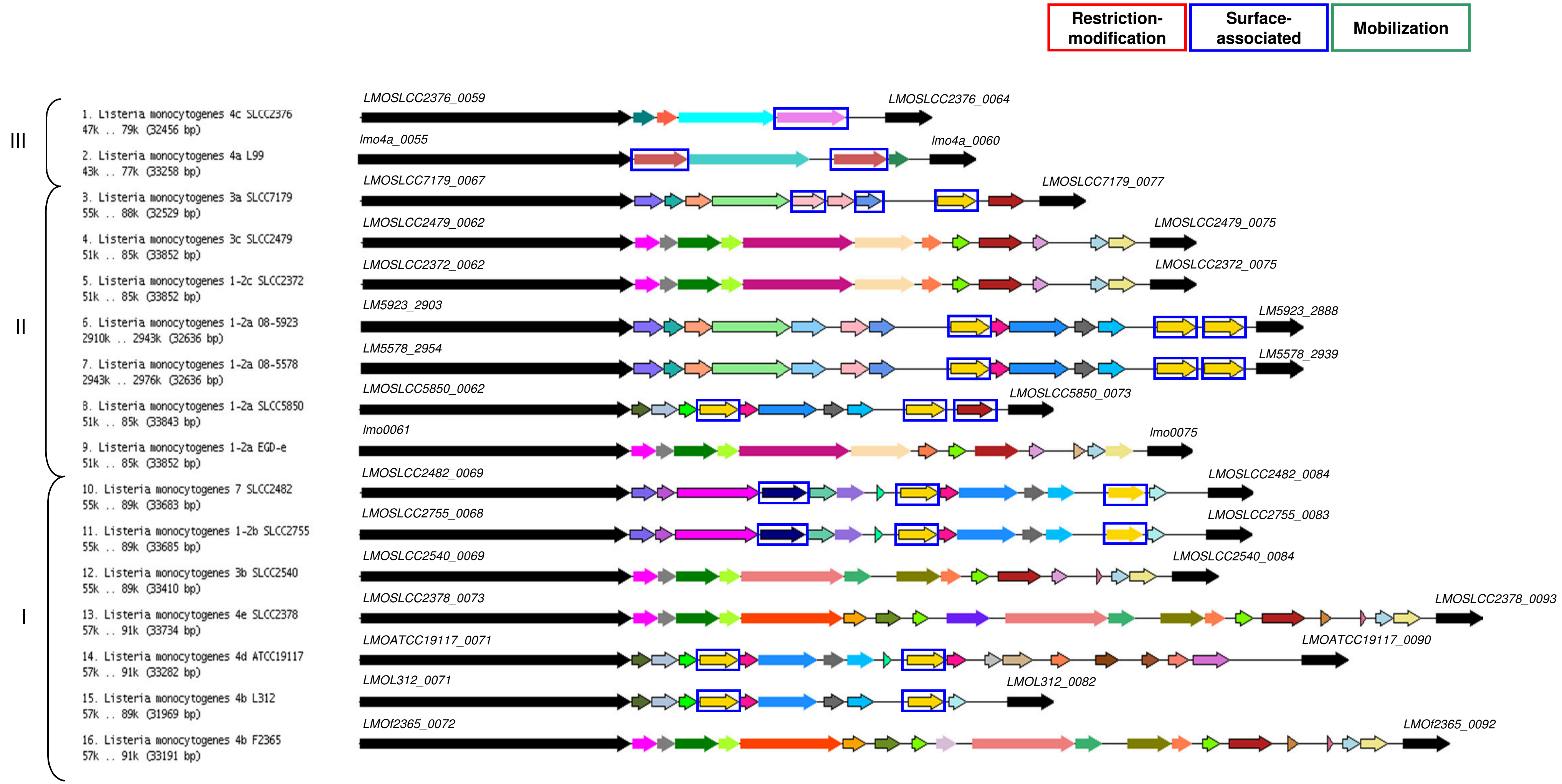

Hypervariable hotspot 2 (lmo0137-lmo0152)

Based on a homology cutoff >60% amino acid identity and >80% coverage. Black arrows indicate mutually conserved core genes. A black border denotes a deviation from the average codon usage of the chromosome. Loci of strains 1/2a 08-5923 and 08-5578 were reversed to enhance clarity.

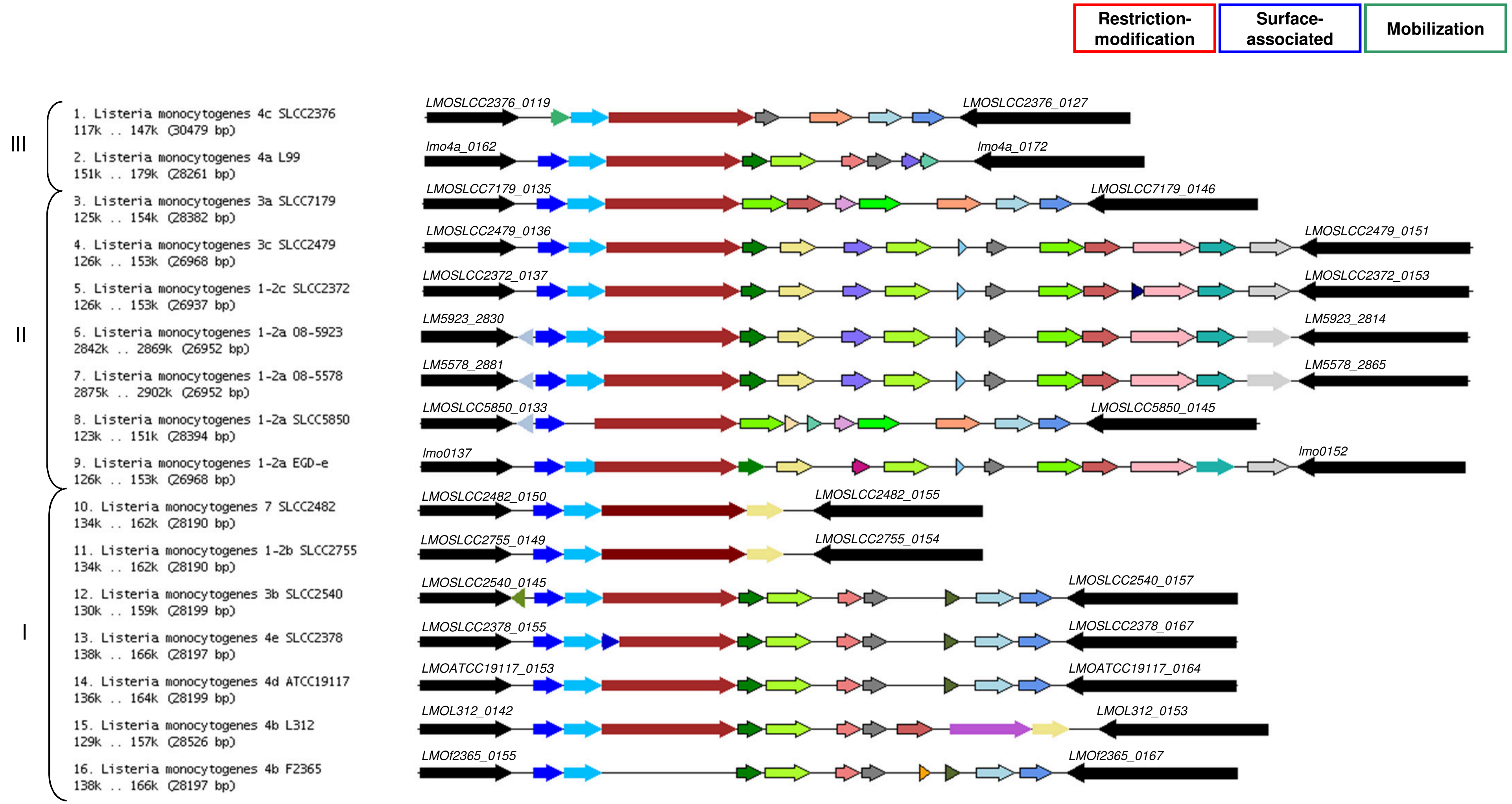

Hypervariable hotspot 3 (lmo0293-lmo0296)

Based on a homology cutoff >60% amino acid identity and >80% coverage. Black arrows indicate mutually conserved core genes. A black border denotes a deviation from the average codon usage of the chromosome.

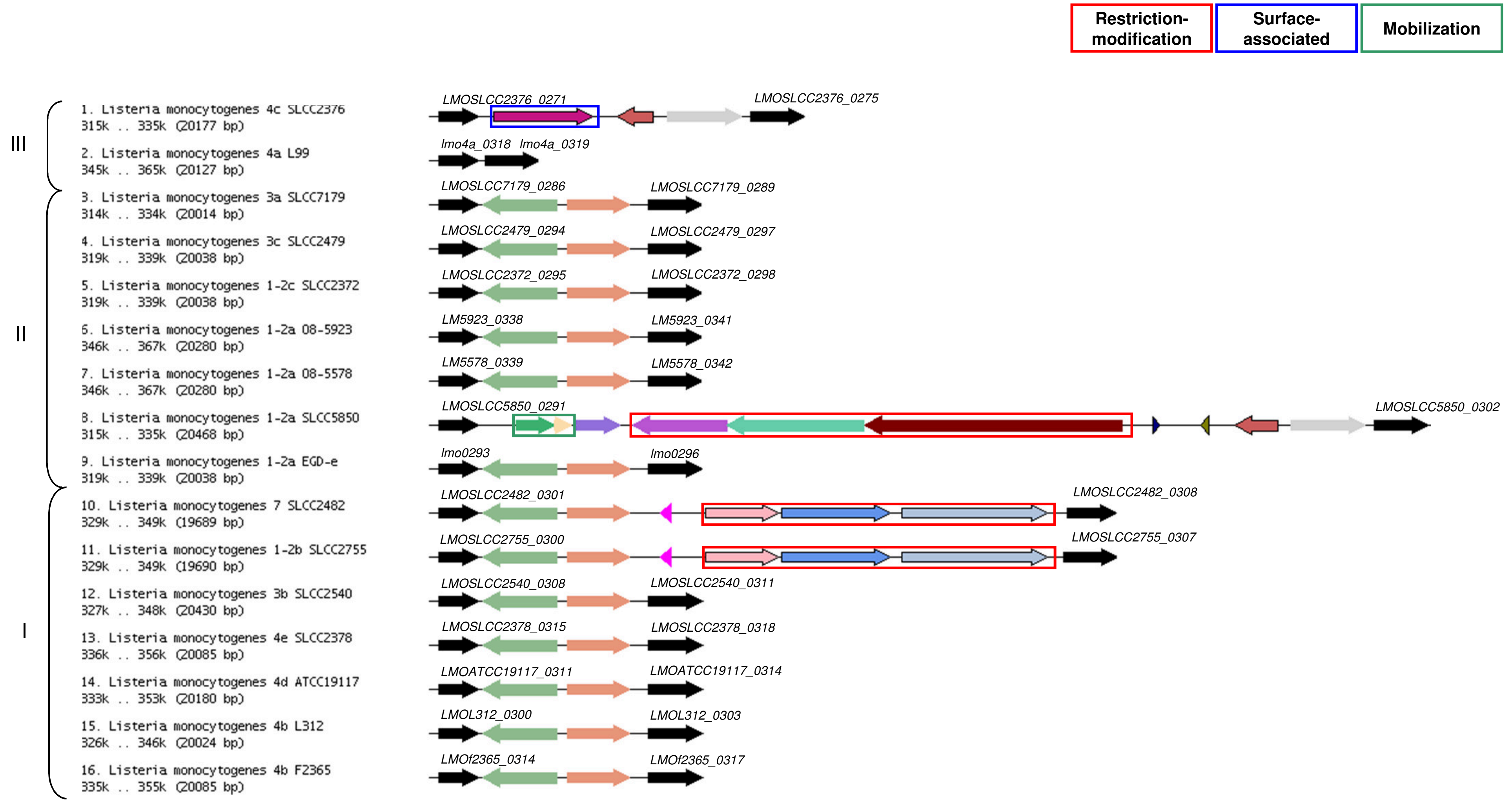

Hypervariable hotspot 4 (lmo0301-lmo0314)

Based on a homology cutoff >60% amino acid identity and >80% coverage. Black arrows indicate mutually conserved core genes. A black border denotes a deviation from the average codon usage of the chromosome.

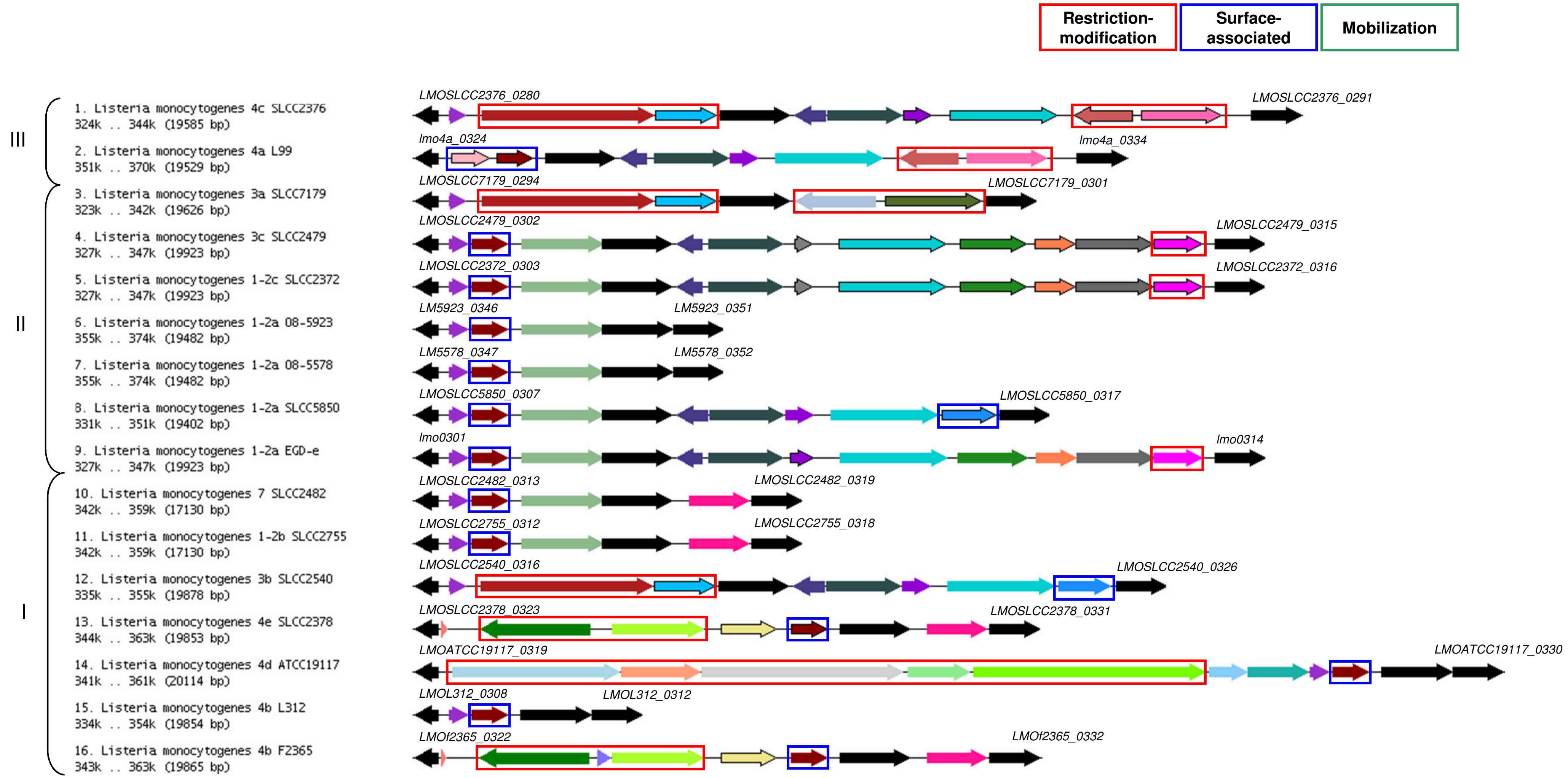

Hypervariable hotspot 5 (lmo0377-lmo0382)

Based on a homology cutoff >60% amino acid identity and >80% coverage. Black arrows indicate mutually conserved core genes. A black border denotes a deviation from the average codon usage of the chromosome.

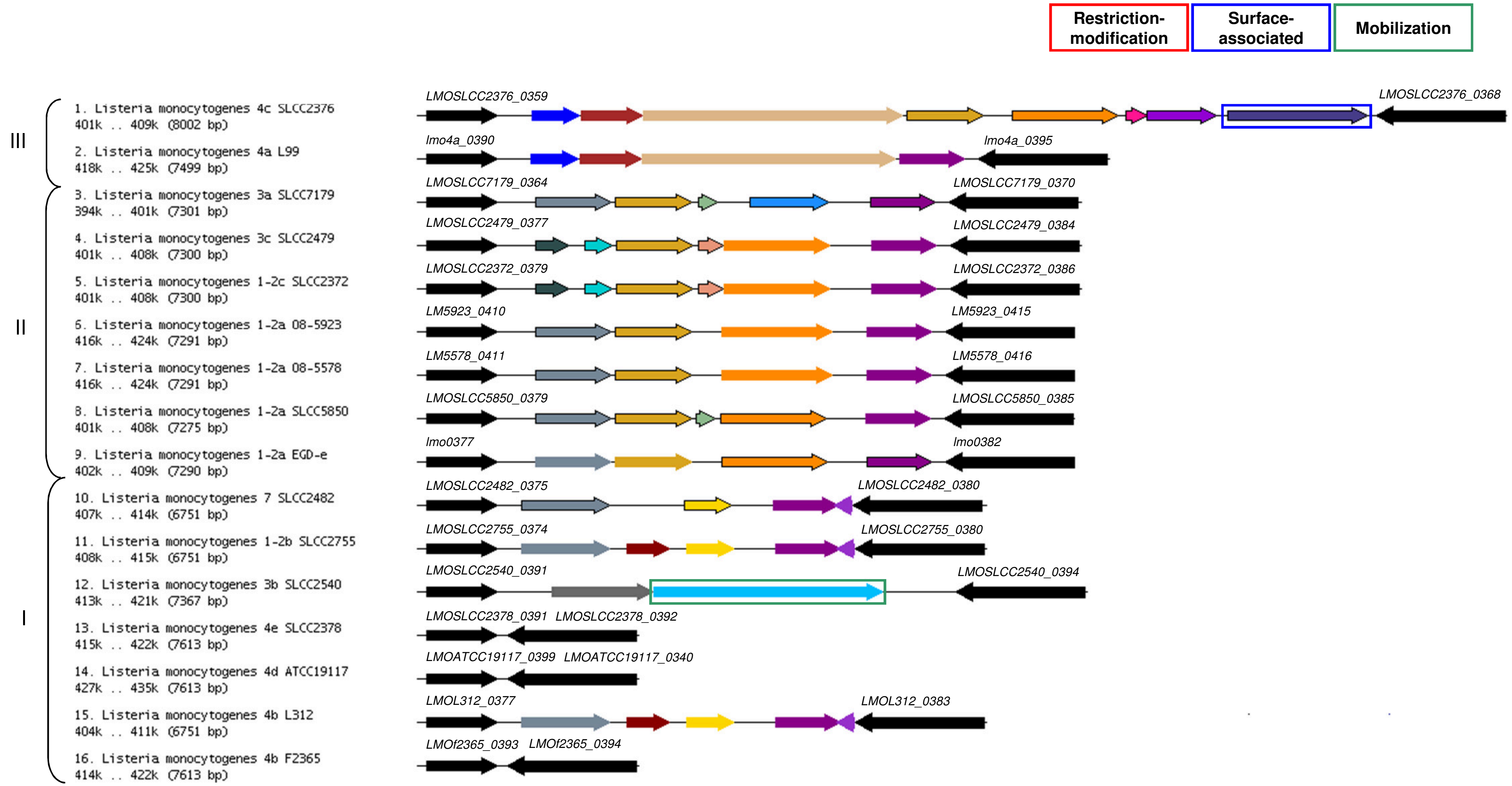

Hypervariable hotspot 6 (lmo0432-lmo0437)

Based on a homology cutoff >60% amino acid identity and >80% coverage. Black arrows indicate mutually conserved core genes. A black border denotes a deviation from the average codon usage of the chromosome.

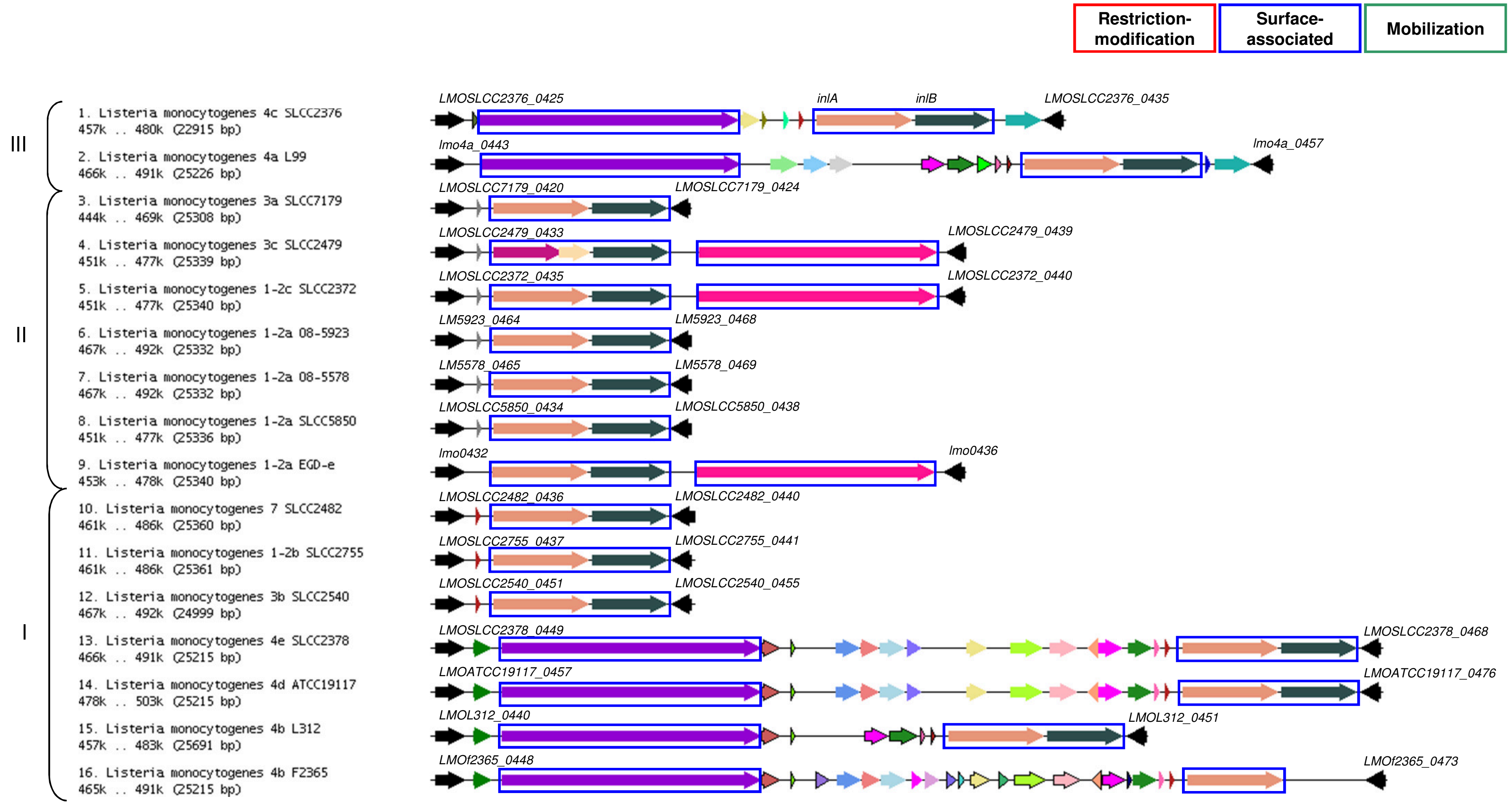

Hypervariable hotspot 7 (lmo0458-lmo0480)

Based on a homology cutoff >60% amino acid identity and >80% coverage. Black arrows indicate mutually conserved core genes. A black border denotes a deviation from the average codon usage of the chromosome.

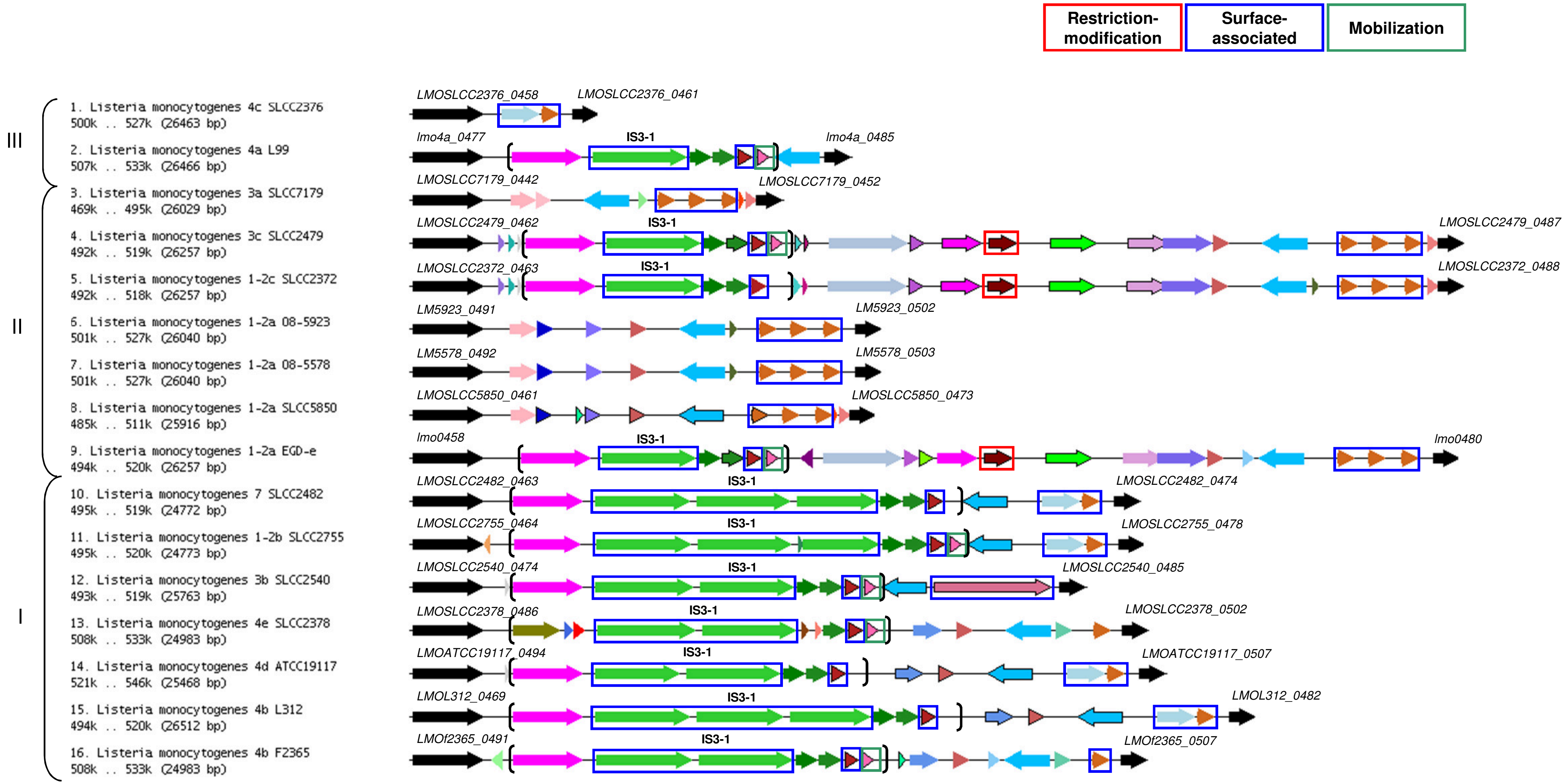

Hypervariable hotspot 8 (lmo1096-lmo1126)

Based on a homology cutoff >60% amino acid identity and >80% coverage. Black arrows indicate mutually conserved core genes. A black border denotes a deviation from the average codon usage of the chromosome.

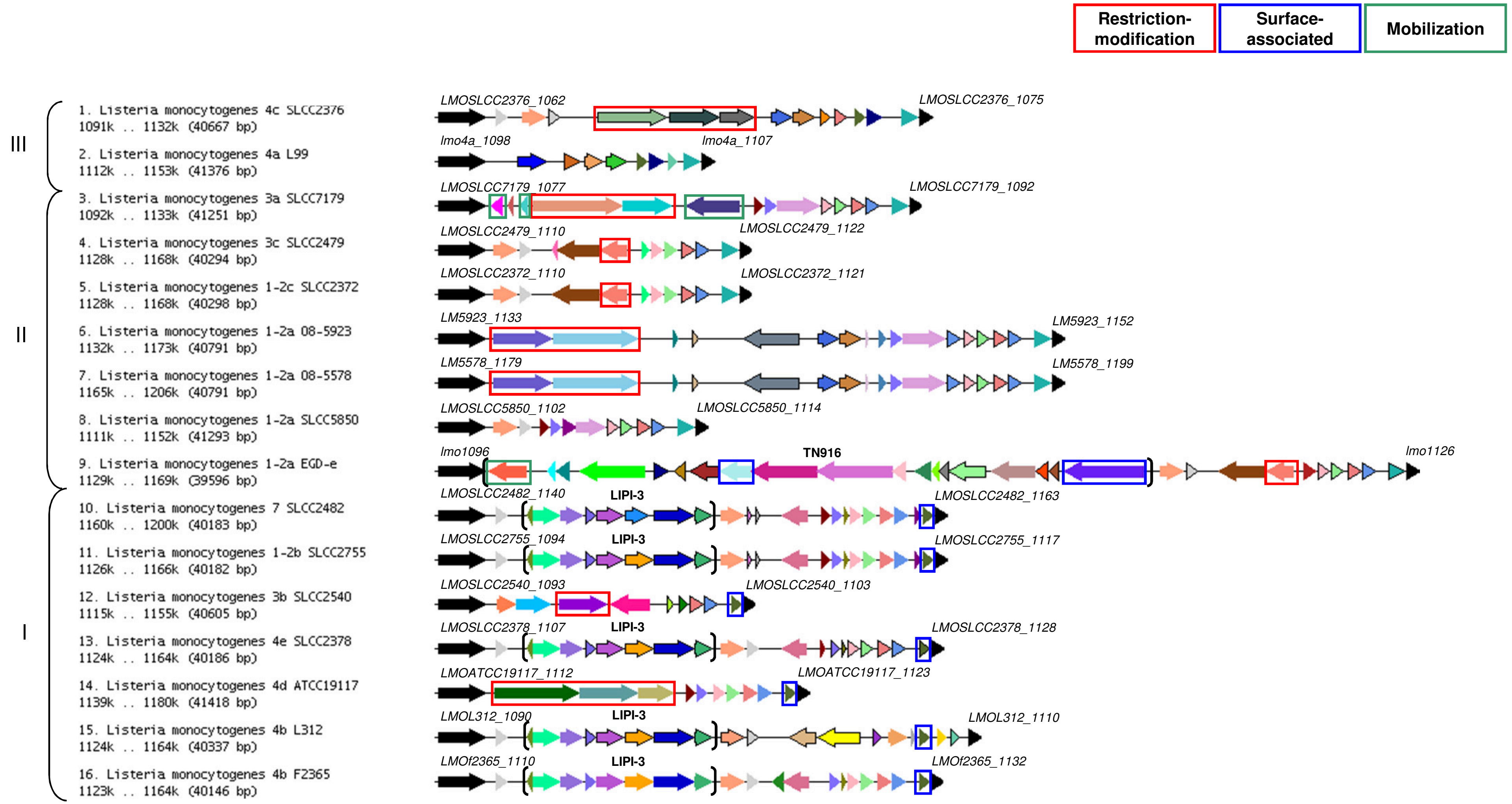

Hypervariable hotspot 9 (lmo2025-lmo2028)

Based on a homology cutoff >60% amino acid identity and >80% coverage. Black arrows indicate mutually conserved core genes. A black border denotes a deviation from the average codon usage of the chromosome.

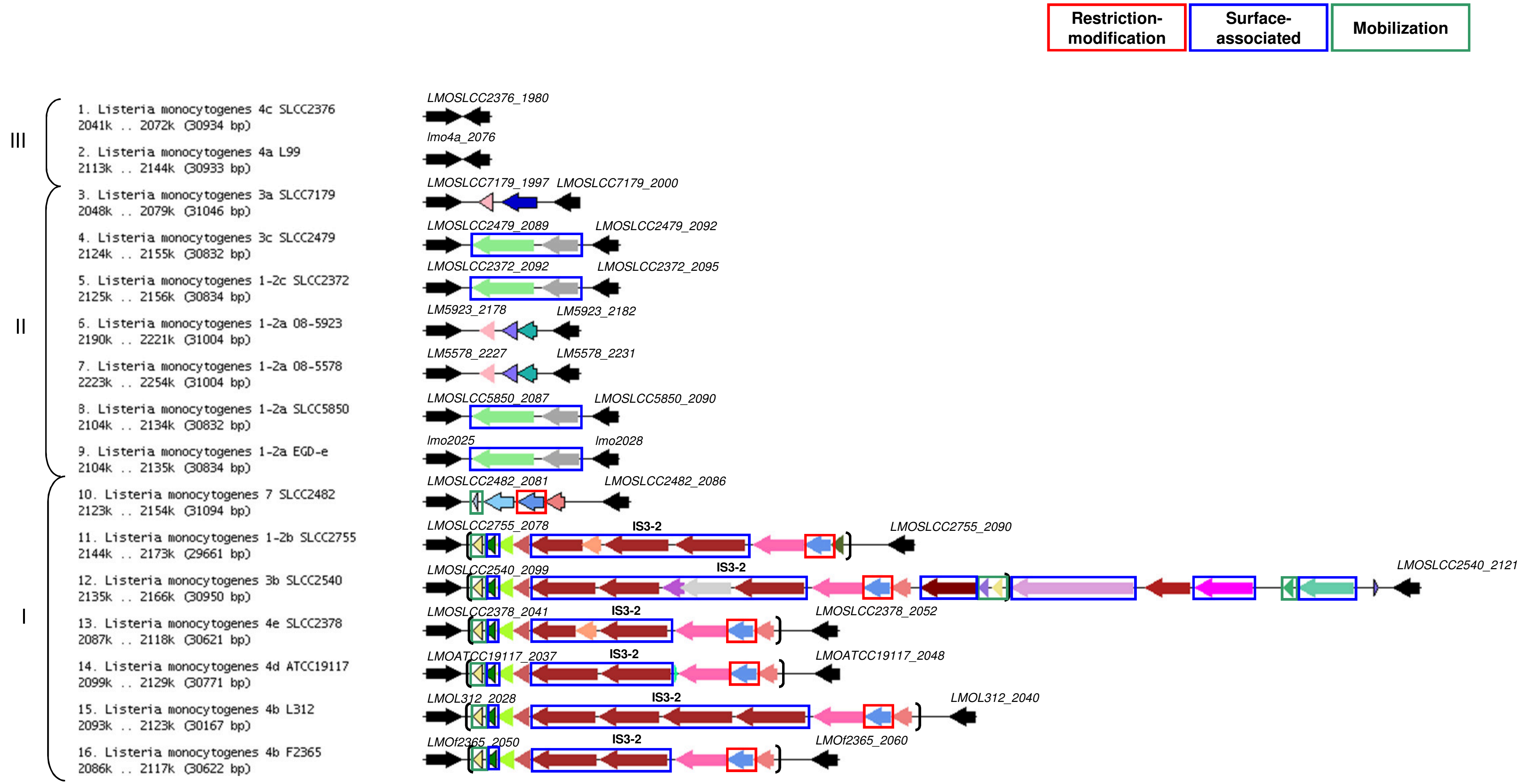

Supplement: Additional file 3 — Comparative genomic GECO figures of hyper variable hotspots. Comparative GECO depictions of insertional hotspots highlighting extensive mosaicism. [file 1471-2164-14-47-S3.pdf]
