# Supplementary material for: Reassessment of the Listeria monocytogenes pan-genome reveals dynamic integration hotspots and mobile genetic elements as major components of the accessory genome
Source: BMC Genomics. 2013 Jan 22;14:47. doi: 10.1186/1471-2164-14-47 (PMC3556495; doi:10.1186/1471-2164-14-47)

Content and conservation of transposons ICELm1 and TN554 in species *L. monocytogenes*

Based on a homology cutoff >60% amino acid identity and >80% coverage. A black border denotes a deviation from the average codon usage of the chromosome.

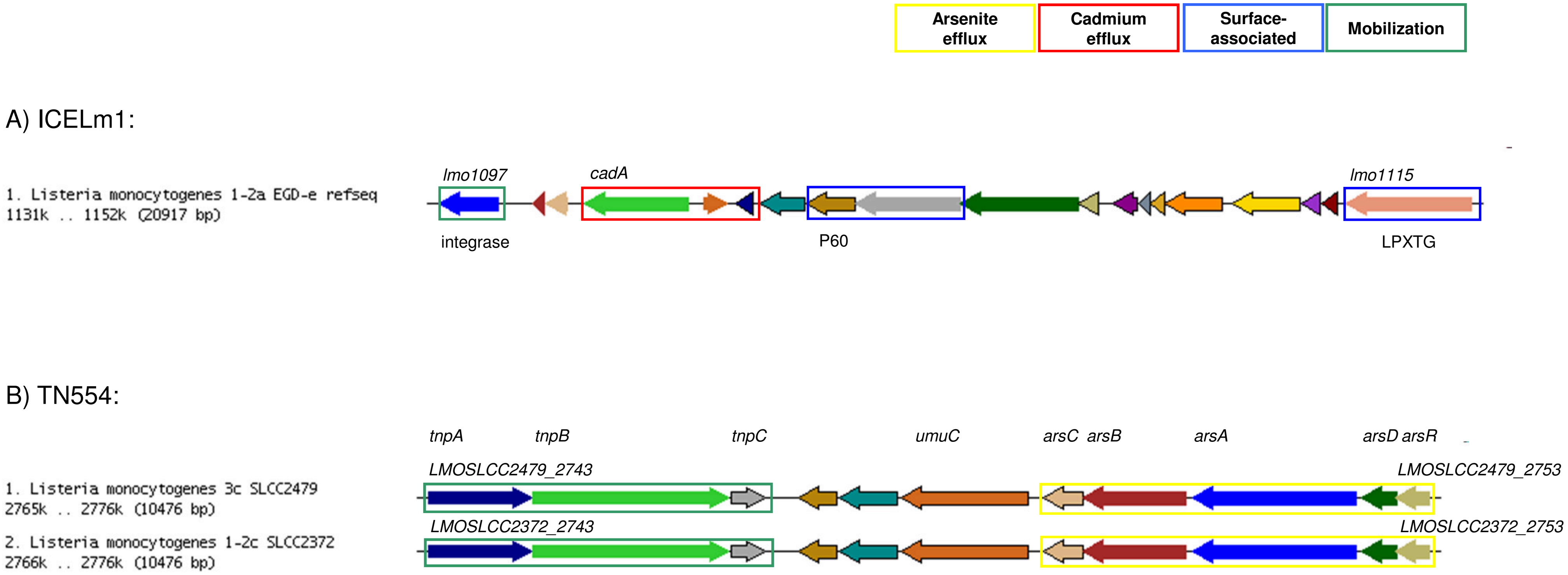

Supplement: Additional file 5 — Comparative genomic GECO figures of transposons ICELm1 and TN554. Comparative GECO depiction using a homology measure of 60% amino acid identity and 80% coverage. Displays content and conservation of two transposons. [file 1471-2164-14-47-S5.pdf]
