# Supplementary material for: Reassessment of the Listeria monocytogenes pan-genome reveals dynamic integration hotspots and mobile genetic elements as major components of the accessory genome
Source: BMC Genomics. 2013 Jan 22;14:47. doi: 10.1186/1471-2164-14-47 (PMC3556495; doi:10.1186/1471-2164-14-47)

Duplication of IS3-like transposon

Based on a homology cutoff >60% amino acid identity and >80% coverage. A black border denotes a deviation from the average codon usage of the chromosome.

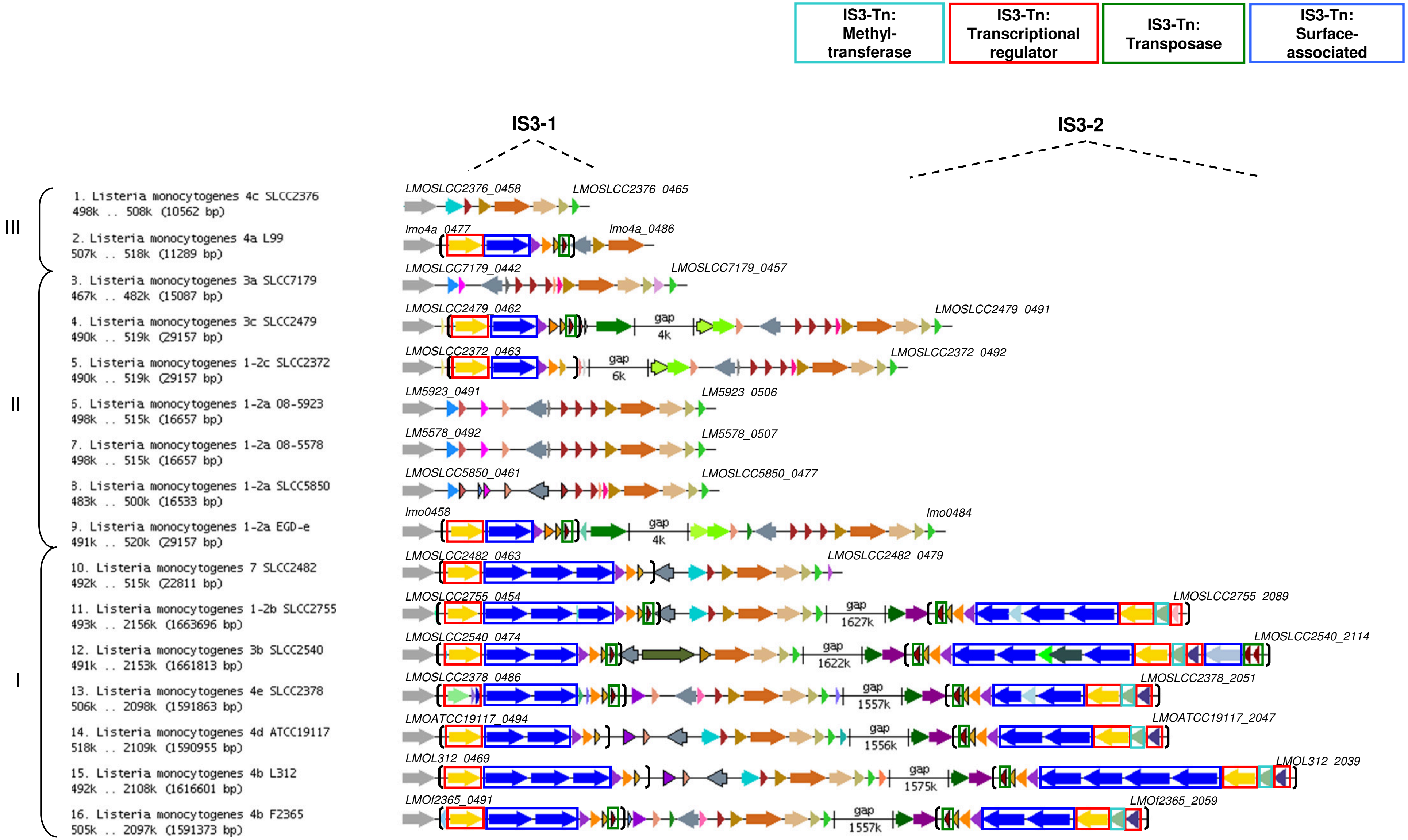

Supplement: Additional file 6 — Comparative genomic GECO figures of IS3 elements. Comparative GECO depiction using a homology measure of 60% amino acid identity and 80% coverage. Displays duplication of IS3-like transposon. [file 1471-2164-14-47-S6.pdf]
