# Supplementary material for: Reassessment of the Listeria monocytogenes pan-genome reveals dynamic integration hotspots and mobile genetic elements as major components of the accessory genome
Source: BMC Genomics. 2013 Jan 22;14:47. doi: 10.1186/1471-2164-14-47 (PMC3556495; doi:10.1186/1471-2164-14-47)

CRISPR/Cas loci 1-3

Based on a homology cutoff >60% amino acid identity and >80% coverage. A black border denotes a deviation from the average codon usage of the chromosome. Locus 3 includes a *trans*-acting sRNA called tracrRNA that was described to compensate for a missing endoribonuclease in conjunction with host factor RNase III [Deltcheva et al., 2011, CRISPR RNA maturation by trans-encoded small RNA and host factor RNase III].

A) Region *lmo0509-lmo0526*

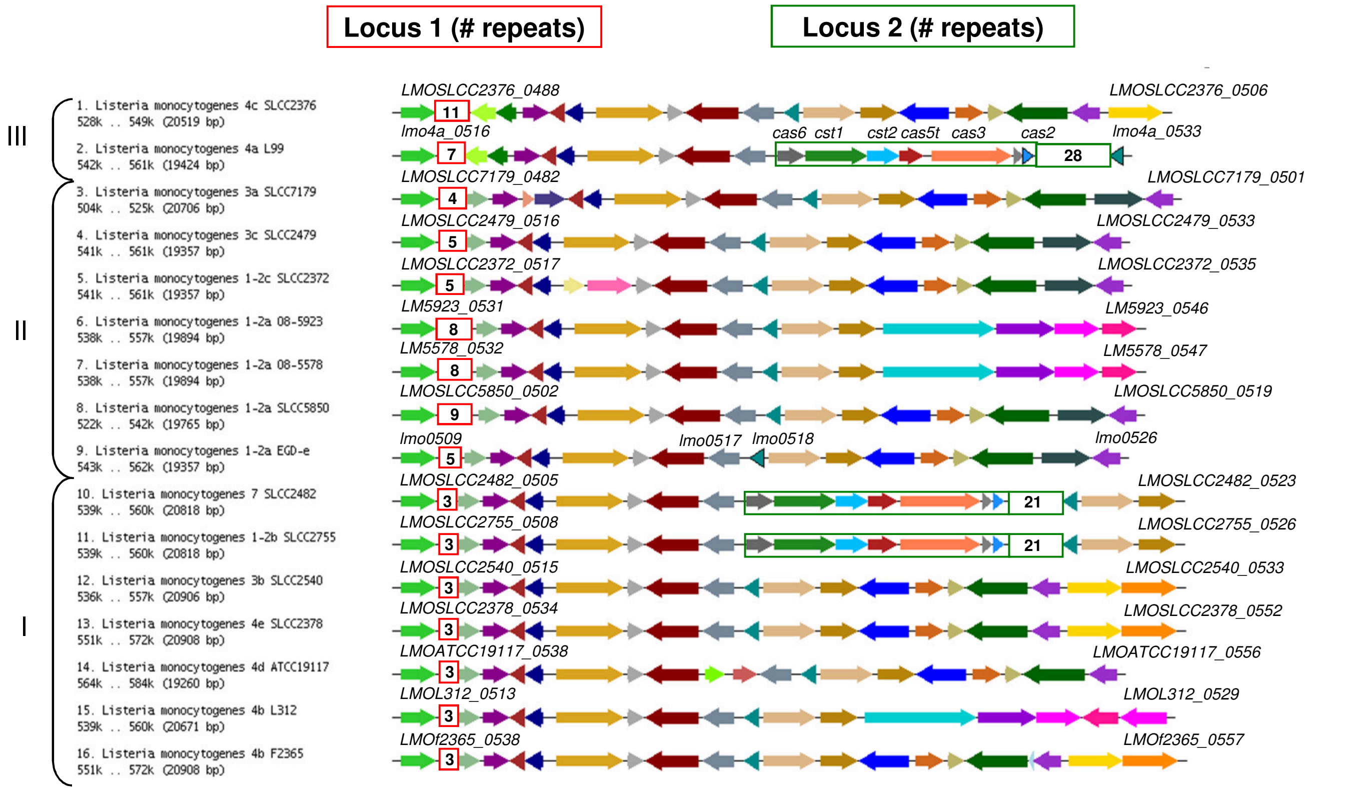

B) Region *lmo2590-lmo2614*

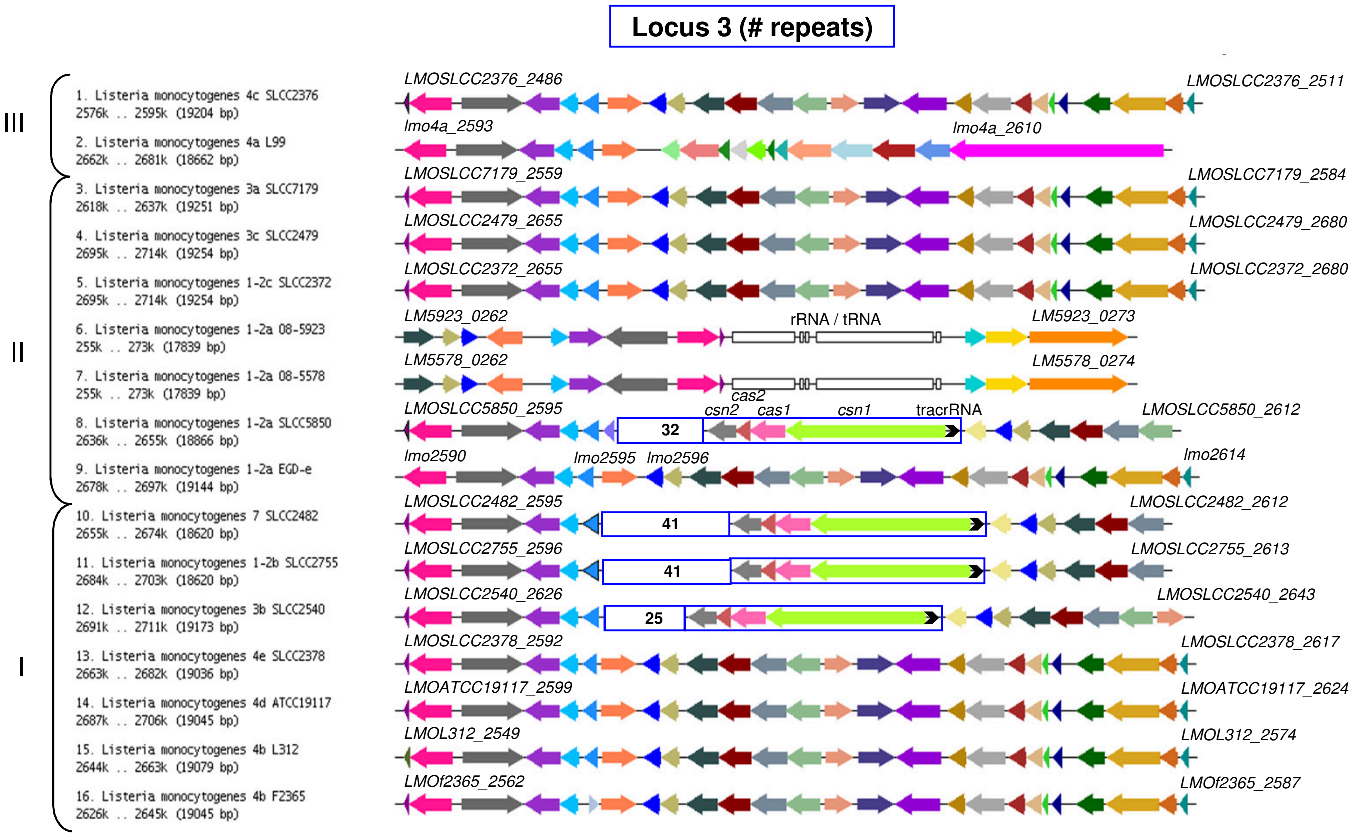

Supplement: Additional file 7 — Comparative genomic GECO figure of CRISPR/Cas loci. Comparative GECO depictions of three CRISPR/Cas loci using a minimum CDS homology measure of 60% amino acid identity and 80% coverage.Cas genes and spacer/repeat arrays are framed. Locus 1 displayed no associated Cas genes. Locus 3 includes a trans-acting sRNA called tracrRNA that was described to compensate for a missing endoribonuclease in conjunction with host factor RNase III. [file 1471-2164-14-47-S7.pdf]
