# Supplementary figures and images for: Reassessment of the Listeria monocytogenes pan-genome reveals dynamic integration hotspots and mobile genetic elements as major components of the accessory genome
Source: BMC Genomics. 2013 Jan 22;14:47. doi: 10.1186/1471-2164-14-47 (PMC3556495; doi:10.1186/1471-2164-14-47)

Growth in BHI medium at 37°C

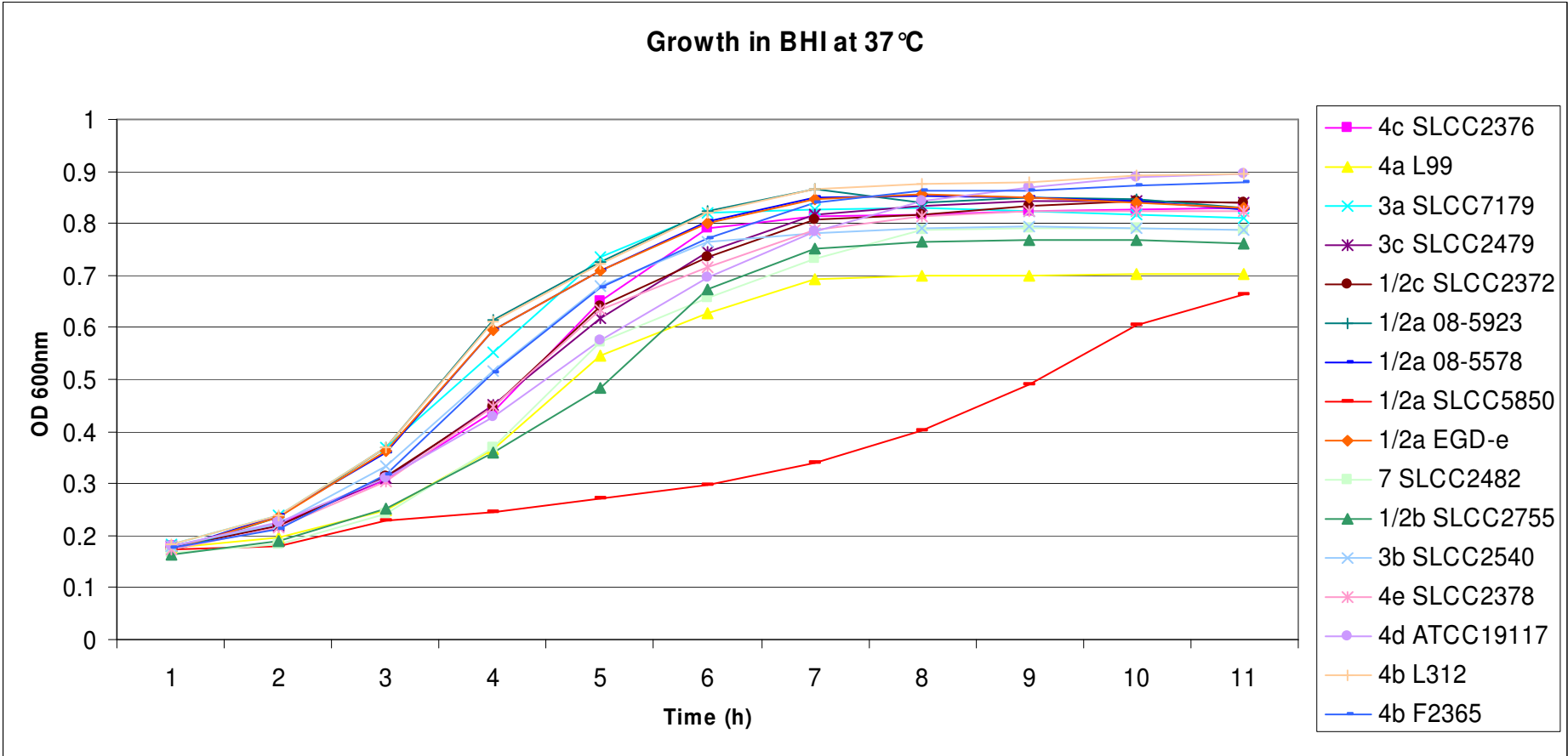

Supplement: Additional file 11 — Growth curves BHI. Growth of L. monocytogenes in BHI medium at 37°C. [file 1471-2164-14-47-S11.pdf]
