# Supplementary material for: Reassessment of the Listeria monocytogenes pan-genome reveals dynamic integration hotspots and mobile genetic elements as major components of the accessory genome
Source: BMC Genomics. 2013 Jan 22;14:47. doi: 10.1186/1471-2164-14-47 (PMC3556495; doi:10.1186/1471-2164-14-47)
Supplement: Additional file 12 — Detailed analyses of reductive evolution of virulence-associated genes. In-depth information about previously described virulence and pathogenicity indicated genes that are absent or truncated in one of the compared strains. [file 1471-2164-14-47-S12.pdf]

Detailed analyses of reductive evolution of virulence-associated genes

Primary virulence genes responsible for invasion of host cells are the virulence gene cluster (*lmo0200-5*) and two internalins (*lmo0433-4*). Among these is the main regulator of virulence *prfA* bearing an N-terminal deletion in 1/2a SLCC5850 and phospholipase *plcA,* necessary for the escape from phagosomes, and showing a premature stop-codon in 3a SLCC7179. Primary virulence genes *inlA* and *inlB* mediate invasion of epithelial cells and a range of other cell types, and display truncations in 3c SLCC2479 and 4b F2365, respectively [1]. Absence of internalin A from the cell wall fraction of strain 3c SLCC2479 was additionally confirmed by immunoblot analysis [2]. These deletions are expected to lead to severe virulence attenuation in the respective strains and were confirmed for strains 1/2a SLCC5850, 3a SLCC7179 and 3c SLCC2479 by low invasion rates of HeLa cells [2]. The deleterious N-terminal mutation of *prfA* in strain SLCC5850 is putatively not responsible for slow growth in BHI medium, as assessed by low degrees of transcription of this gene in this condition, as well as isogenic *prfA* deletion mutants [3]. Instead, growth attenuation of 1/2a SLCC5850 may result from the specific absence of 12 genes found in all other compared strains, coding for various proteins involved in energy production/conversion and metabolism.

One of the repeats of the *actA* gene, which promotes intracellular movement, was previously found to be deleted in the majority of strains of serotype 1/2b and 4b including strains 4b F2365 and 4a L99 [4]. This deletion is also exhibited by strains 3b SLCC2540, 4b L312 and 4e SLCC2378, likely resulting in a limited effect on phenotype.

A range of genes facilitate the attachment to and invasion of host cells. All compared strains of lineage I with the exception of 3b SLCC2540 and 4d ATCC19117 contain the LIPI-3 Listeriolysin S module (*LMOf2365_1113-9*), which translates into a bacteriocin-like haemolytic virulence factor present in strains that caused the majority of epidemics [5]. Strain 7 SLCC2482 shows a premature stop-codon in the bacteriocin biosynthesis cyclohydratase (homologue of *LMOf2365_1117).* Considering the phylogenetic distribution, LLS was putatively included in an ancestral strain of lineage I and lost in 3b SLCC2540 and 4d ATCC19117 either during separate events or in an ancestral strain of both. Surface proteins Vip and Auto are required for efficient entry into various eukaryotic cell lines [6,7]. The gene encoding Vip is absent from lineage III and all serotype 1/2a strains with the exception of EGD-e, while *aut* could not be found in any serogroup 4 strain, indicating either a reduced range of target cells or a non-homologous replacement in the respective strains. Indeed, all serogroup 4 strains contain a specific amidase gene located at the same relative chromosomal position, which may be able to substitute this functionality. Autolysin Ami can break covalent bonds in the peptidoglycan of cell walls and is involved in binding to host cells by means of GW repeat domains as described for strain EGD-e [8]. Strain 7 SLCC2482 displays a premature stop-codon in the *ami* gene.

As already described, strains of lineage III and especially strain 4a L99 have lost a number of internalins (*inlFC* / *inlFCGHEIJ*), reducing the range of cell types that can be infected, as well as further abilities for adhesion [4,9,10].Interestingly, we identified two distinct versions of *inlJ* in the other strains: variant 1 (2751 bp) is present in lineage I with the exception of 4b L312, variant 2 (~2550 bp) exists in 4b L312 and lineages II/III, respectively. Variant 2 differs from 1 by a central indel of 71

bp, resulting in the absence of one of five intestinal mucus binding protein domains (MucBP) [11]. The pattern of distribution of *inlJ* implies the presence of the variant containing four MucBP domains in a *L. monocytogenes* ancestor, followed by duplication of one domain in most strains of lineage I. Taken together, this putatively leads to differing adhesion characteristics of *inlJ* towards eukaryotic cells when comparing the majority of strains of lineage I to II/III.

Another class of genes attends to general stress response regulated by alternative sigma factor B (*sigB*) that is itself regulated by multiple other proteins [12-14]. Among these are *rsbS, rsbV* and *rsbU,* which contain premature stop-codons in 1/2c SLCC2372, 4d ATCC19117 and 3c SLCC2479, respectively. Affected strains will putatively hold a misregulated *sigB* regulon and thus decreased stress resistance during infection and in the environment. The bile tolerance locus (*lmo0752-4*) and stress survival islet 1 (SSI-1, *lmo0444-8*) are implicated in protecting the bacterium in the gastro-intestinal system and gall bladder [15,16]. Strain 4c SLCC2376 putatively lost the former system including gene *btlB*, while compared strains of serotypes 4a, 3a, 3b, 4e, 4d, and 4b do not harbor the latter. All strains do contain homologues of the bile salt hydrolase gene (*bsh*, *lmo2067*) and the bile exclusion locus (*lmo1421-2*) indicating that acid resistance in the deleterious strains is likely diminished but not absent [17]. Furthermore, the complete arginine metabolic pathway (*lmo0036-41*) is absent from lineage III. It is regulated by *sigB* and *prfA,* implied in acid tolerance and described as a virulence factor in the murine model [18].

Eukaryotic hosts are described to express a range of cationic antimicrobial peptides that can be countered by bacteria by changing the cell wall composition to include different lipoteichoic acids (eg. *dltA, lmo0974*) [19,20]. *DltA* is part of the regulon of virulence regulator *virR* in *L. monocytogenes* and contains a premature stop-codon in

1/2a SLCC5850, putatively leading to increased sensitivity towards cationic antimicrobial peptides [21].

It was previously demonstrated, that 21 genes were specifically differentially regulated inside IFN-γ-activated macrophages, considered to be the primary host defense effector cells, in comparison to non-activated macrophages [22]. A putatively secreted protein (*lmo0478*) and a protein kinase (*lmo0618*) were not mutually conserved in all compared strains. The former was found to be absent from strain 4a L99 and most strains of lineage I, while the latter shows a C-terminal truncation in strain 1/2b SLCC2755. The functions of these genes have still to be elucidated, but their absence may hamper the ability of the respective strains to survive extreme stresses inside activated macrophages and thus their ability to proliferate inside a host.

MogR (*lmo0674*) was identified in tissue culture models as a motility gene repressor which downregulates *flaA* in the intracellular niche to avoid immune system detection [23]. Strain 3a SLCC7179 displays a premature stop-codon in this gene, putatively leading to increased recognition by the host and thus decreased virulence.

Recently, a study was published describing a secreted virulence factor called LntA (*lmo0438*) targeting the chromatin repressor BAHD1 in the host cell nucleus to activate interferon stimulated genes and thus control bacterial colonization of the host [24]. This gene is absent from serotype 4a (L99 and HCC23) and apathogenic strains of other species being *L. innocua* Clip11262, *L. welshimeri* SLCC5334 and *L. seeligeri* SLCC3954. A nucleotide alignment of *lmo0438* and the corresponding regions in 4a L99 and HCC23 revealed the presence of sequence remnants (stretch of 117 bp with ca. 97% nucleotide identity) indicating a deletion of this gene in an ancestral strain of serotype 4a, putatively contributing to impaired growth of strains of serotype 4a in the host.

Two-component response regulator gene *agrA* (*lmo0051*) was shown to influence the production of several secreted proteins leading to reduced virulence of a deletion mutant in the mouse model [25]. This gene exhibits a premature stop-codon in strain 4e SLCC2378, bearing implications for the virulence of this strain.

The differing availability of nutrients in the environment and in the eukaryotic host necessitates a meta- and catabolic shift for facultative parasitic microbes. Glycerol kinase *glpK2* (*lmo1034*) is a significant member of glycerol catabolism of intracellularly growing *L. monocytogenes* EGD-e in Caco2-cells and was found to be absent from strain 4a L99 [26].

Most compared strains of serotype 1/2a with the exception of EGD-e either showed a low invasion rate of Caco-2 cells (08-5578, 08-5923) or were completely unable to enter this type of cells (SLCC5850) (data not shown). We identified genes related to attachment and invasion that were present in EGD-e and absent from all other compared strains of serotype 1/2a (internalin *lmo1289*, virulence factor *vip*), as well as specific deletions in strains 08-5578/5923 (internalins *lmo0801, lmo2026, lmo2027*), and SLCC5850 (primary virulence regulator *prfA*, phage holin *lmo2279*), that may explain the inability of respective strains to efficiently invade Caco-2 cells. These data indicate that a correlation of Caco-2 invasion rates between strains of the same serotype is not self-evident in support of previous observations [27].

References

1. Chen Y, Ross WH, Whiting RC, Van SA, Nightingale KK, Wiedmann M, Scott VN: **Variation in *Listeria monocytogenes* dose responses in relation to**

subtypes encoding a full-length or truncated internalin A. *Appl Environ Microbiol* 2011, **77:**1171-1180.

2. Chatterjee SS, Otten S, Hain T, Lingnau A, Carl UD, Wehland J, Domann E, Chakraborty T: **Invasiveness is a variable and heterogeneous phenotype in *Listeria monocytogenes* serotype strains.** *Int J Med Microbiol* 2006, **296:**277-286.

3. Zhou Q, Feng F, Wang L, Feng X, Yin X, Luo Q: **Virulence regulator PrfA is essential for biofilm formation in *Listeria monocytogenes* but not in *Listeria innocua*.** *Curr Microbiol* 2011, **63:**186-192.

4. Hain T, Ghai R, Billion A, Kuenne CT, Steinweg C, Izar B, Mohamed W, Mraheil MA, Domann E, Schaffrath S et al.: **Comparative genomics and transcriptomics of lineages I, II, and III strains of *Listeria monocytogenes*.** *BMC Genomics* 2012, **13:**144.

5. Cotter PD, Draper LA, Lawton EM, Daly KM, Groeger DS, Casey PG, Ross RP, Hill C: **Listeriolysin S, a novel peptide haemolysin associated with a subset of lineage I *Listeria monocytogenes*.** *PLoS Pathog* 2008, **4:**e1000144.

6. Cabanes D, Sousa S, Cebria A, Lecuit M, Garcia-del PF, Cossart P: **Gp96 is a receptor for a novel *Listeria monocytogenes* virulence factor, Vip, a surface protein.** *EMBO J* 2005, **24:**2827-2838.

7. Cabanes D, Dussurget O, Dehoux P, Cossart P: **Auto, a surface associated autolysin of *Listeria monocytogenes* required for entry into eukaryotic cells and virulence.** *Mol Microbiol* 2004, **51:**1601-1614.

8. Milohanic E, Jonquieres R, Glaser P, Dehoux P, Jacquet C, Berche P, Cossart P, Gaillard JL: **Sequence and binding activity of the autolysin-adhesin Ami from epidemic *Listeria monocytogenes* 4b.** *Infect Immun* 2004, **72:**4401-4409.

9. Raffelsbauer D, Bubert A, Engelbrecht F, Scheinpflug J, Simm A, Hess J, Kaufmann SH, Goebel W: **The gene cluster inlC2DE of *Listeria monocytogenes* contains additional new internalin genes and is important for virulence in mice.** *Mol Gen Genet* 1998, **260:**144-158.

10. Doumith M, Cazalet C, Simoes N, Frangeul L, Jacquet C, Kunst F, Martin P, Cossart P, Glaser P, Buchrieser C: **New aspects regarding evolution and virulence of *Listeria monocytogenes* revealed by comparative genomics and DNA arrays.** *Infect Immun* 2004, **72:**1072-1083.

11. Linden SK, Bierne H, Sabet C, Png CW, Florin TH, McGuckin MA, Cossart P: ***Listeria monocytogenes* internalins bind to the human intestinal mucin MUC2.** *Arch Microbiol* 2008, **190:**101-104.

12. Kang CM, Brody MS, Akbar S, Yang X, Price CW: **Homologous pairs of regulatory proteins control activity of *Bacillus subtilis* transcription factor sigma(b) in response to environmental stress.** *J Bacteriol* 1996, **178:**3846-3853.

13. Palma M, Cheung AL: **sigma(B) activity in *Staphylococcus aureus* is controlled by RsbU and an additional factor(s) during bacterial growth.** *Infect Immun* 2001, **69:**7858-7865.

14. Chaturongakul S, Boor KJ: **RsbT and RsbV contribute to sigmaB-dependent survival under environmental, energy, and intracellular stress conditions in *Listeria monocytogenes*.** *Appl Environ Microbiol* 2004, **70:**5349-5356.

15. Begley M, Sleator RD, Gahan CG, Hill C: **Contribution of three bile-associated loci, bsh, pva, and btlB, to gastrointestinal persistence and bile tolerance of *Listeria monocytogenes*.** *Infect Immun* 2005, **73:**894-904.

16. Ryan S, Begley M, Hill C, Gahan CG: **A five-gene stress survival islet (SSI-1) that contributes to the growth of *Listeria monocytogenes* in suboptimal conditions.** *J Appl Microbiol* 2010, **109:**984-995.

17. Sleator RD, Wemekamp-Kamphuis HH, Gahan CG, Abee T, Hill C: **A PrfA-regulated bile exclusion system (BilE) is a novel virulence factor in *Listeria monocytogenes*.** *Mol Microbiol* 2005, **55:**1183-1195.

18. Ryan S, Begley M, Gahan CG, Hill C: **Molecular characterization of the arginine deiminase system in *Listeria monocytogenes*: regulation and role in acid tolerance.** *Environ Microbiol* 2009, **11:**432-445.

19. Harris F, Dennison SR, Phoenix DA: **Anionic antimicrobial peptides from eukaryotic organisms.** *Curr Protein Pept Sci* 2009, **10:**585-606.

20. Kovacs M, Halfmann A, Fedtke I, Heintz M, Peschel A, Vollmer W, Hakenbeck R, Bruckner R: **A functional dlt operon, encoding proteins required for incorporation of d-alanine in teichoic acids in gram-positive bacteria, confers resistance to cationic antimicrobial peptides in *Streptococcus pneumoniae*.** *J Bacteriol* 2006, **188:**5797-5805.

21. Mandin P, Fsihi H, Dussurget O, Vergassola M, Milohanic E, Toledo-Arana A, Lasa I, Johansson J, Cossart P: **VirR, a response regulator critical for *Listeria monocytogenes* virulence.** *Mol Microbiol* 2005, **57:**1367-1380.

22. Mraheil MA, Billion A, Mohamed W, Rawool D, Hain T, Chakraborty T: **Adaptation of *Listeria monocytogenes* to oxidative and nitrosative stress in IFN-gamma-activated macrophages.** *Int J Med Microbiol* 2011, **301:**547-555.

23. Grundling A, Burrack LS, Bouwer HG, Higgins DE: ***Listeria monocytogenes* regulates flagellar motility gene expression through MogR, a transcriptional repressor required for virulence.** *Proc Natl Acad Sci U S A* 2004, **101:**12318-12323.

24. Lebreton A, Lakisic G, Job V, Fritsch L, Tham TN, Camejo A, Mattei PJ, Regnault B, Nahori MA, Cabanes D et al.: **A bacterial protein targets the BAHD1 chromatin complex to stimulate type III interferon response.** *Science* 2011, **331:**1319-1321.

25. Autret N, Raynaud C, Dubail I, Berche P, Charbit A: **Identification of the agr locus of *Listeria monocytogenes*: role in bacterial virulence.** *Infect Immun* 2003, **71:**4463-4471.

26. Joseph B, Mertins S, Stoll R, Schar J, Umesha KR, Luo Q, Muller-Altrock S, Goebel W: **Glycerol metabolism and PrfA activity in *Listeria monocytogenes*.** *J Bacteriol* 2008, **190:**5412-5430.

27. Jaradat ZW, Bhunia AK: **Adhesion, invasion, and translocation characteristics of *Listeria monocytogenes* serotypes in Caco-2 cell and mouse models.** *Appl Environ Microbiol* 2003, **69:**3640-3645.
