# Supplementary material for: Reassessment of the Listeria monocytogenes pan-genome reveals dynamic integration hotspots and mobile genetic elements as major components of the accessory genome
Source: BMC Genomics. 2013 Jan 22;14:47. doi: 10.1186/1471-2164-14-47 (PMC3556495; doi:10.1186/1471-2164-14-47)

Number of surface-associated proteins

Domains were predicted by Augur (HMM evalue<10, score>5) [Billion at al., 2006, Augur - a computational pipeline for whole genome microbial surface protein prediction and classification].

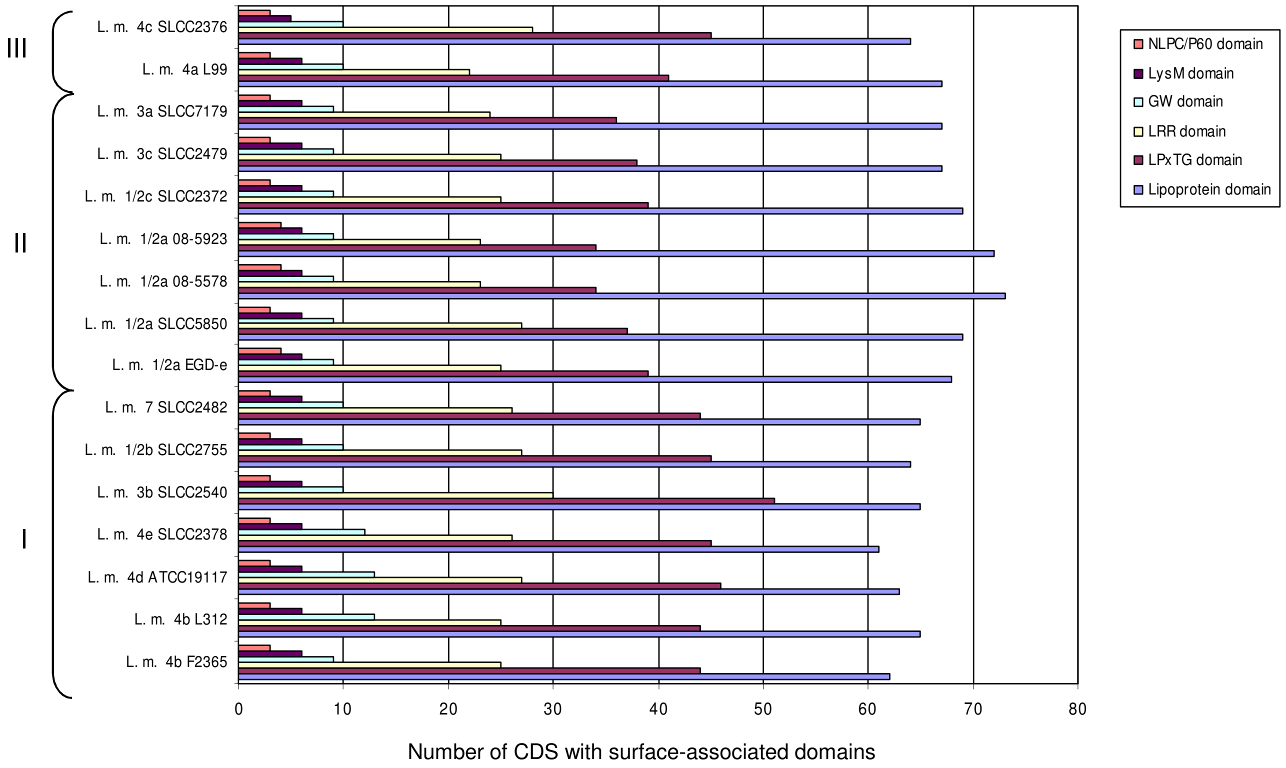

Supplement: Additional file 13 — Plot of Surface-associated CDS. Bar plot depicting the distribution of all surface-associated protein coding genes among studied strains. [file 1471-2164-14-47-S13.pdf]
