# Supplementary material for: Reassessment of the Listeria monocytogenes pan-genome reveals dynamic integration hotspots and mobile genetic elements as major components of the accessory genome
Source: BMC Genomics. 2013 Jan 22;14:47. doi: 10.1186/1471-2164-14-47 (PMC3556495; doi:10.1186/1471-2164-14-47)
Supplement: Additional file 14 — Distribution of surface-associated genes displays conserved lineage-backbones with strain-specific adaptations. Detailed analysis of presence and absence of surface-associated genes. [file 1471-2164-14-47-S14.pdf]

Distribution of surface-associated genes displays conserved lineage-backbones with strain-specific adaptations

A variety of cell wall or membrane anchored genes are important for the survival of strains of species *Listeria monocytogenes* by mediating interaction with the environment and the infected host. The following analysis will highlight relevant patterns of presence or absence of surface-associated genes and suggest evolutionary explanations.

The first class to be discussed are genes containing P60 and LysM domains that were described to be involved in the invasion of host cells (P60, P45), degradation of the bacterial cell wall and various enzymatic or binding activities [1]. These show little variation among the studied chromosomes, bearing 3-4 and 5-6 genes, respectively.

Known functions of lipoproteins include substrate binding for ABC transporter systems and adhesion, deployed to promote survival in the mouse model (OppA) and to mediate entry into eukaryotic cells (LpeA) [2-4]. Studied strains contain a minimum of 61 lipoprotein coding genes (4e SLCC2378) and a maximum of 73 (1/2a 08-5578) including 57 mutually conserved genes. Those showing a differential distribution rarely correlate with lineages or serotypes, but display strain-specific patterns implying relatively recent insertions. Non-core lipoproteins were frequently located in chromosomal hotspots of horizontal gene transfer and found inside or adjacent to prophage insertions. Bacteriophages employ lipoproteins for various tasks including adhesion, membrane fusion and receptor binding [5,6]. We also identified an IS3-like transposon (*lmo0459-64*) containing a putative lipoprotein (*lmo0460*) with weak RGD/LRR repeats, which was overrepresented in epidemic lineage I (*LMOf2365_2051-7*), suggesting further research considering a relation to virulence or pathogenicity.

A putative transposition moved two adjacent lipoproteins (*lmo1264-5*), conserved at the same relative chromosomal position in lineages I and II, by ca. 600kb in lineage III where they may have reinserted in a reversed position (*LMOSLCC2376_1746-5*). An ancestor of lineage III putatively harbored both genes at the same location as lineages I and II, since a C-terminal stretch of *lmo1265*(56 bp, 86% identity) can still be found here. A BLASTP search directed versus the NCBI nr database revealed that only chromosomes of genus *Listeria* contain homologues of these genes, indicating that this sequence was either transposed inside the same chromosome or deleted and laterally reinserted from another *Listeria* strain. Lineages I and II contain internalin C (*lmo1786*) at the same locus which displays the reinserted lipoproteins in lineage III. Since no remnant sequence of *inlC* can be found in lineage III, it is unclear if this gene existed in a common ancestor of *L. monocytogenes*. But considering the evolutionary trend towards reduction of virulence as illustrated by lineage III, we propose that *inlC* was commonly conserved, leading to an evolutionarily neutral or negative region for lineage III, which resulted in the deletion of *inlC* and the putative transposition of the two lipoproteins to this locus.

We furthermore analyzed the distribution of genes anchored to the bacterial surface by interaction with lipoteichoic acids using a GW-motif, most of which were previously implicated in the adhesion to or invasion of eukaryotic cells [7,8]. Compared strains contain 9-13 genes showing a GW-domain including seven core genes. Among the differentially distributed genes are those coding for Auto (*lmo1076*) and Ami (*lmo2558*) that were found to be absent or mutated in strains of serogroup 4, respectively. We also identified a module of ca. 2kb in all strains of serogroup 4 of lineage I, which inserted between homologues of genes *lmo0012-lmo0013* in reference strain EGD-e. It bears 2-3 GW-domain genes that display varying sizes and high partial homologies, indicating an ongoing deleterious process and putatively dysfunctional proteins. All strains of epidemic lineage I show another exclusive gene (*LMOf2365_1974*) with both LPXTG and GW

domains which may become a future research target considering cell wall anchored modulators of virulence or pathogenicity.

The final type of surface-associated genes to be discussed in this study are internalins implied in cell adhesion and invasion of host cells [9]. While all internalins contain a leucine-rich repeat (LRR) domain indicated in protein-protein interaction, the majority furthermore show a signal peptide that tags the respective protein for the secretory pathway [10]. We found that all but four putative internalin gene clusters contain at least one homologue with an identifiable signal peptide, three-fourths include an LPXTG anchor motif (34/42) and six are putatively secreted. GW or WxL anchors were only identified inside one internalin each. We additionally searched for presence of an InlB B-repeat (68bp consensus, identity > 50%, coverage > 65%) which was proposed to bind a further host cell receptor [11]. This sequence seems to be a hallmark of previously described virulence-associated internalins, as only *inlI, inlC* and *inlJ* do not contain at least one copy. An InlB B-repeat was found in 15 clusters, thus increasing the probability of the respective genes to be involved in host-pathogen interaction.

The distribution of candidate internalins shows a relatively homogeneous pattern for strains of lineage I, while lineage II and III are more heterogeneous. Only four of 42 homology clusters are mutually conserved, confirming previous observations of diversity [12]. On the other hand, we could only identify nine genes that were not mutually conserved inside at least one lineage using relaxed homology criteria (identity >50%, coverage >40%), indicating that early lineage-ancestors already contained most of the internalin prototypes, that were adapted for specific needs in the following evolutionary period as previously described.

A number of known virulence-associated internalins were absent in a subset of strains, putatively resulting in a reduced number of infectable cell types (lineage III: *inlC* and *inlF*, 4a L99: *inlGHE*, *inlI* and *inlJ*, 3c SLCC2479: *inlA*, 4b F2365: *inlB*).Interestingly, we identified two different

versions of *inlF* and *inlJ* in lineage I compared to lineages II/III, putatively resulting in different adhesion properties.

There are two described versions of an internalin cluster (*lmo0263-4*) putatively resulting from recombination events in an early ancestor [9]. Strains 1/2a EGD-e, 3c SLCC2479, and 1/2c SLCC2372 contain the *inlGHE* module, while all other strains display the *inlC2DE* variant [13]. An exception to this are strain 4a L99 that has lost the complete *inlGHE* cluster and 4d ATCC19117, which contains a premature stop-codon in *inlD*. Both variants contain genes putatively regulated by sigB and thus may contribute to bacterial survival following stress [14].

We detected a 3b SLCC2540-specific module including four putative internalin genes containing both LRR and LPXTG motifs adjacent to multiple IS3 family transposases (*LMOSLCC2540_2112-9*). The internalin genes and intermittently located transposases display partial homologies, respectively (data not shown) indicating a common evolutionary background that may include duplication and/or recombination events.

Furthermore, a putative ancient double duplication or recombination of LPXTG-anchored internalin *lmo1290* was identified, which is presumed to have happened inside ancestral strains of lineages. Lineage I contains only homologues of gene *lmo1290*, lineage II harbors an additional weak homologue *lmo1289* (43% amino acid identity, 99% coverage), while lineage III shows two more variants of this gene (*lmo4a_1344-5*) with a comparable degree of similarity. A comparison to non-pathogenic species of genus *Listeria* showed that only *L. innocua* Clip11262 contained a homologue of *lmo1289* (*lin1328*) while *L. welshimeri* SLCC5334 and *L. seeligeri* SLCC3954 displayed no internalin-like gene at this relative position, indicating a possible association to pathogenicity. No impact on phenotype has been described for these genes yet.

Only one internalin was found to be specific and mutually conserved for lineage I (*LMOf2365_0805*), apart from a central deletion of 200 bp in the homologue of strain 7 SLCC2482, indicating this gene for further research regarding virulence.

Taken together, we found that most surface-associated genes are either mutually conserved or were likely present in an early ancestor of a lineage, implying a fixed core-functionality that is rarely complemented by strain-specific additions. Of all surveyed classes, internalins seemed to be the most diverse, driven by duplication, recombination and transposition. We furthermore identified a large number of novel surface-associated genes, including their distribution among all serotypes of species *L. monocytogenes*, opening a pool of candidates for future analysis considering virulence and pathogenicity.

References

1. Cabanes D, Dehoux P, Dussurget O, Frangeul L, Cossart P: **Surface proteins and the pathogenic potential of *Listeria monocytogenes*.** *Trends Microbiol* 2002, **10:**238-245.

2. Glaser P, Frangeul L, Buchrieser C, Rusniok C, Amend A, Baquero F, Berche P, Bloecker H, Brandt P, Chakraborty T et al.: **Comparative genomics of *Listeria* species.** *Science* 2001, **294:**849-852.

3. Borezee E, Pellegrini E, Berche P: **OppA of *Listeria monocytogenes*, an oligopeptide-binding protein required for bacterial growth at low temperature and involved in intracellular survival.** *Infect Immun* 2000, **68:**7069-7077.

4. Reglier-Poupet H, Pellegrini E, Charbit A, Berche P: **Identification of LpeA, a PsaA-like membrane protein that promotes cell entry by *Listeria monocytogenes*.** *Infect Immun* 2003, **71:**474-482.

5. Bryl K, Kedzierska S, Laskowska M, Taylor A: **Membrane fusion by proline-rich Rz1 lipoprotein, the bacteriophage lambda Rz1 gene product.** *Eur J Biochem* 2000, **267:**794-799.

6. Hong J, Kim KP, Heu S, Lee SJ, Adhya S, Ryu S: **Identification of host receptor and receptor-binding module of a newly sequenced T5-like phage EPS7.** *FEMS Microbiol Lett* 2008, **289:**202-209.

7. Jonquieres R, Pizarro-Cerda J, Cossart P: **Synergy between the N- and C-terminal domains of InlB for efficient invasion of non-phagocytic cells by *Listeria monocytogenes*.** *Mol Microbiol* 2001, **42:**955-965.

8. Bierne H, Cossart P: ***Listeria monocytogenes* surface proteins: from genome predictions to function.** *Microbiol Mol Biol Rev* 2007, **71:**377-397.

9. Bierne H, Sabet C, Personnic N, Cossart P: **Internalins: a complex family of leucine-rich repeat-containing proteins in *Listeria monocytogenes*.** *Microbes Infect* 2007, **9:**1156-1166.

10. Kobe B, Kajava AV: **The leucine-rich repeat as a protein recognition motif.** *Curr Opin Struct Biol* 2001, **11:**725-732.

11. Ebbes M, Bleymuller WM, Cernescu M, Nolker R, Brutschy B, Niemann HH: **Fold and function of the InlB B-repeat.** *J Biol Chem* 2011, **286:**15496-15506.

12. Tsai YH, Orsi RH, Nightingale KK, Wiedmann M: ***Listeria monocytogenes*** **internalins are highly diverse and evolved by recombination and positive selection.** *Infect Genet Evol* 2006, **6:**378-389.

13. Raffelsbauer D, Bubert A, Engelbrecht F, Scheinpflug J, Simm A, Hess J, Kaufmann SH, Goebel W: **The gene cluster inlC2DE of *Listeria monocytogenes* contains additional new internalin genes and is important for virulence in mice.** *Mol Gen Genet* 1998, **260:**144-158.

14. Kazmierczak MJ, Mithoe SC, Boor KJ, Wiedmann M: ***Listeria monocytogenes*** **sigma B regulates stress response and virulence functions.** *J Bacteriol* 2003, **185:**5722-5734.
