# Supplementary material for: Reassessment of the Listeria monocytogenes pan-genome reveals dynamic integration hotspots and mobile genetic elements as major components of the accessory genome
Source: BMC Genomics. 2013 Jan 22;14:47. doi: 10.1186/1471-2164-14-47 (PMC3556495; doi:10.1186/1471-2164-14-47)

Transposition of lipoproteins *lmo1264-5* in lineage III into locus that putatively contained *inlC* previously

Based on a homology cutoff >60% amino acid identity and >80% coverage. A black border denotes a deviation from the average codon usage of the chromosome.

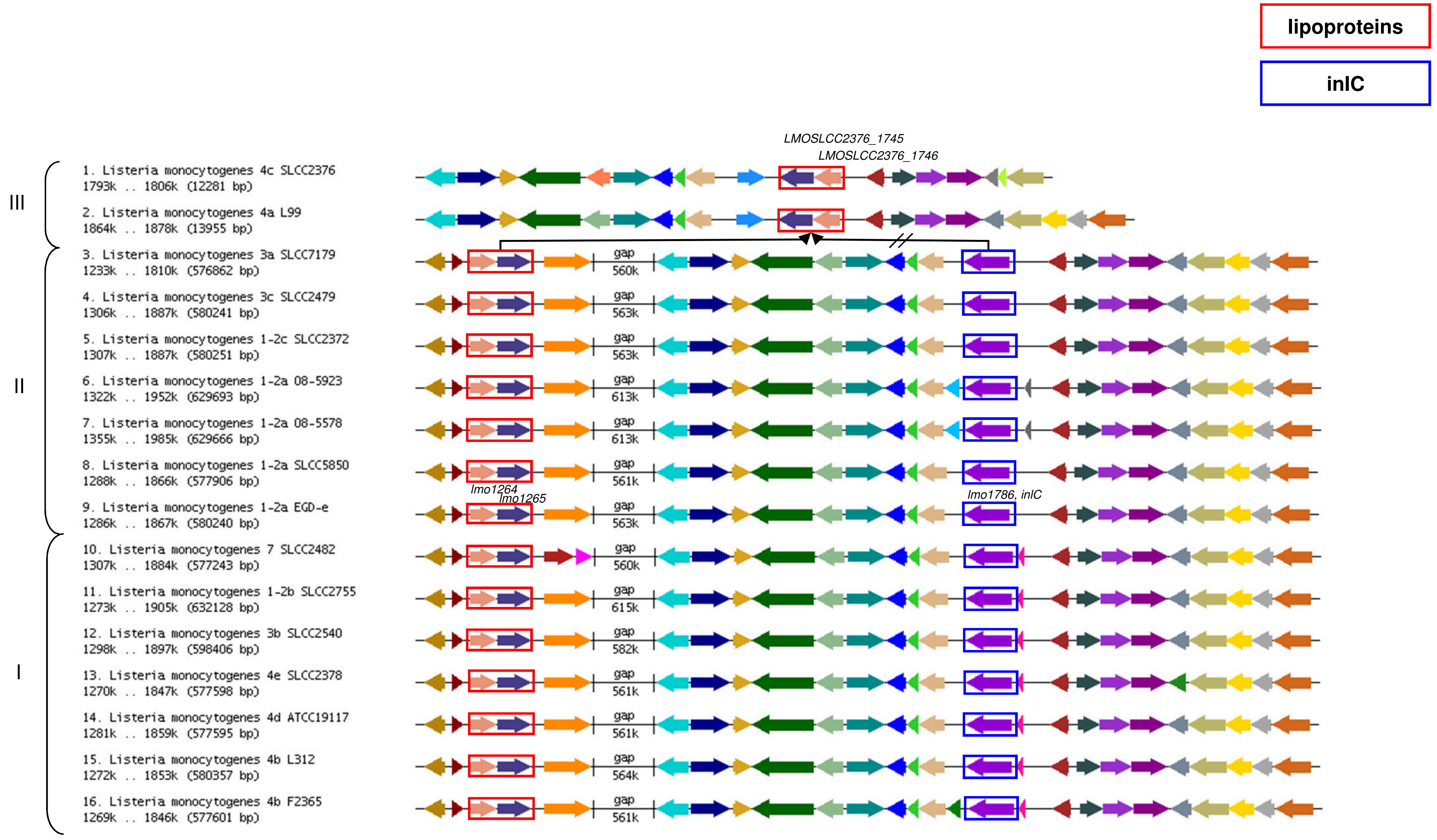

Supplement: Additional file 17 — Putative transposition of lipoproteins lmo1264-5 in lineage III. Comparative GECO depiction using a homology measure of 80% amino acid identity and 90% coverage. Displays the putative transposition of lipoproteins lmo1264-5 in lineage III into the locus that putatively held inlC previously. [file 1471-2164-14-47-S17.pdf]
